# A probiotic bacterium modulates antitumour γδ T-cell responses in lung cancer

**DOI:** 10.3389/fimmu.2026.1750569

**Published:** 2026-03-24

**Authors:** Yoshihiko Goto, Garry Dolton, Hannah Thomas, Théo Morin, Yuka Tajima, Kosuke Imamura, Shinya Sakata, Kentaro Oka, Atsushi Hayashi, Motomichi Takahashi, Takamasa Ueno, Takuro Sakagami, Yusuke Tomita, Andrew K. Sewell, Chihiro Motozono

**Affiliations:** 1 Division of Infection and Immunity, Joint Research Center for Human Retrovirus Infection, Kumamoto University, Kumamoto, Japan; 2 Department of Respiratory Medicine, Faculty of Life Sciences, Kumamoto University, Kumamoto, Japan; 3 Division of Infection and immunity Cardiff University School of Medicine, Cardiff, United Kingdom; 4 R&D Division, Miyarisan Pharmaceutical Co., Ltd., Saitama, Japan; 5 Systems Immunity Research Institute, Cardiff University, Cardiff, United Kingdom

**Keywords:** butyrophilin 3A, *Clostridium butyricum* (CBM588), gamma delta (γδ) T-cells, immune checkpoint inhibitors (ICIs), microbiome, non-small cell lung cancer (NSCLC), probiotic, Vγ9Vδ2 T-cells

## Abstract

The link between the intestinal microflora and cancer outcomes has been recognized for over a decade. Several recent studies have demonstrated that the gut microbiome is associated with the efficiency of T-cell checkpoint blockade therapy for cancer, raising interest in strategies to harness this effect via consumption of live microorganisms (probiotics). The probiotic *Clostridium butyricum* strain MIYAIRI 588 (CBM588) improves response rates and overall survival in patients receiving immune checkpoint inhibitor (ICI) therapy for non-small cell lung cancer and metastatic renal cell carcinoma but the mechanism underlying this benefit remains unclear. Here, we show that CBM588 spores induce a population of Vγ9Vδ2 T-cells from the peripheral blood of healthy donors and lung cancer patients. A subset of these T-cells responded to, and directly lysed, cancer cell lines via a butyrophilin 3A-dependent mechanism. In patients taking CBM588 alongside checkpoint blockade, using samples from a cohort of 38 patients, peripheral blood Vδ2^+^ T-cells expressed the activation marker CD69 more frequently than in those receiving checkpoint blockade alone and the frequency of Vδ2^+^CD69^+^ cells increased following initiation of CBM588 treatment (p = 0.0041). Pleural effusions from patients receiving ICI with CBM588, although available from only three individuals, also showed a notable shift in the local γδ T-cell compartment from the expected Vδ1 dominance towards Vδ2 cells, suggesting altered recruitment or retention of Vδ2 cells at the tumour site. Across the patient cohort, higher post-treatment frequencies of CD69^+^ Vδ2 T-cells were associated with improved survival and more favourable clinical outcomes. These findings provide a potential mechanism by which manipulation of the intestinal microflora might contribute to cancer prognosis through effects on immune effector cells with intrinsic anticancer properties.

## Introduction

Many cancers are associated with an altered composition of commensal microbiota, raising debate over whether this dysbiosis is a cause or effect of malignancy. Evidence for a causal role has emerged from gnotobiotic mouse models, which show that the microbiota can profoundly influence cancer susceptibility and progression (1). Proposed mechanisms include modulation of systemic and tumour-associated immune responses through microbial metabolites and microbial-derived signalling molecules, as well as effects on antigen presentation and inflammatory pathways. Early animal studies demonstrated that the microbiome modulates the anticancer efficacy of cytotoxic agents such as cyclophosphamide and oxaliplatin (2, 3). More recently, multiple studies have shown that the gut microbiome also impacts the effectiveness of T-cell checkpoint blockade therapy across several cancer types (4–7). The strong association between the gut microbiota and cancer treatment outcomes has resulted in fervent interest in harnessing this effect through the use of probiotics (8). A notable example is *Clostridium butyricum* MIYAIRI 588 (CBM588), an anaerobic probiotic. In retrospective studies at Kumamoto University Hospital, CBM588 was administered alongside checkpoint blockade therapy for non-small cell lung cancer (NSCLC), resulting in significantly improved progression-free survival (PFS; 250 vs. 101 days, *p* = 0.009) and overall survival (OS; median not reached vs. 361 days, *p* = 0.005) compared to patients not receiving the probiotic (9, 10). Additional support comes from a randomized phase 1 trial at City of Hope Medical Centre (NCT03829111), in which daily CBM588 improved response rates and extended PFS in patients with metastatic renal cell carcinoma (mRCC) treated with nivolumab and ipilimumab from 2.5 months to over one year (*p* = 0.001) (11). A second prospective study in mRCC (NCT05122546) reported significantly enhanced objective response rates (*p* = 0.01) and improved six-month PFS in patients receiving CBM588 alongside cabozantinib and nivolumab (12), generating further enthusiasm for this approach.

These clinical observations have increased interest in understanding how probiotics such as CBM588 might influence antitumour immunity.

γδ T-cells are unconventional T-lymphocytes that recognise non-peptide antigens independently of classical major histocompatibility complex (MHC) presentation. In human peripheral blood, the Vγ9Vδ2 subset predominates and responds to small phosphorylated metabolites derived from microbial and tumour isoprenoid pathways, whereas Vδ1^+^ T-cells are enriched at epithelial and tissue sites and recognise a broader set of stress-associated ligands. Vγ9Vδ2 T-cells possess intrinsic cytotoxic capacity and have been implicated in tumour immunosurveillance (13). We hypothesized that CBM588 might activate unconventional anticancer T-cells. Here, we show that CBM588 probiotic tablets containing *Clostridium butyricum* spores and purified spores alone induced a population of Vγ9Vδ2 T-cells from peripheral blood mononuclear cells (PBMCs) of healthy donors from the UK and Japan. A subset of these Vδ2^+^ T-cells responded to a lung cancer cell line via a butyrophilin 3A (BTN3A)-dependent mechanism and were capable of lysing multiple cancer cell lines *in vitro*. Notably, in a retrospective clinical cohort, the activation status of peripheral Vγ9Vδ2 T-cells measured by CD69 expression was higher in lung cancer patients taking CBM588 than in those receiving checkpoint blockade alone, and the frequency of Vδ2^+^CD69^+^ cells increased following initiation of CBM588. These results suggest a mechanism by which microbial probiotics can directly stimulate anticancer T-cell responses and potentially improve cancer prognosis.

## Materials and methods

### Study approval

For the use of human specimens, all protocols involving human subjects recruited at Cardiff University and Kumamoto University were reviewed and approved by the Institutional Review Boards. Donors recruited via the Welsh Blood Service gave informed consent as part of the donation procedure and samples used under local ethical approval granted by the School of Medicine Research Ethics Committee (reference 18/56). Donors recruited in Japan gave their consent and the study was approved by Kumamoto University (approval number 1825).

### Patients

38 patients with NSCLC who received ICIs alone or ICIs + CBM588 (a dose of 120 mg orally three times daily) at Kumamoto University Hospital between 2017 and 2023 were enrolled in this study for *ex vivo* and *in vitro* cytometric analyses (approval number 1825). All human subjects provided written informed consent. Patient overview in **Supplementary Figure 1**.

### Isolation of PBMCs from whole blood

Blood samples from healthy donors were sourced from the Welsh Blood Service (Velindre NHS Trust, Wales, UK) as EDTA treated ‘buffy coats’ and ethical approval granted by the School of Medicine Research Ethics Committee (reference 18/56). Each buffy coat was seronegative for HIV-1, HBV and HCV. Blood and cells derived thereof were handled in accordance with Cardiff University guidelines in alignment with the United Kingdom Human Tissue Act 2004. Blood from the WBS was diluted 1:1 with R10 (RPMI-1640 supplemented with 10% heat-inactivated FBS, 100 U/mL penicillin, 100 µg/mL streptomycin and 2 mM L-glutamine (all Merck, New Jersey, US)) and placed on a Cole-Parmer Stuart™ roller-mixer overnight at 9–11 rpm and room temperature. The following morning the blood was further diluted 2:1 (blood:RPMI-1640) then PBMCs separated using conventional density gradient centrifugation with Sigma-Aldrich Histopaque 1077 (Merck). Red blood cells were removed using lysis buffer (155 mM NH_4_Cl, 10 mM KHCO_3_ and 0.1 mM EDTA, pH7.2-7.4) for 10 min at 37°C. PBMCs were used fresh for culture, without cryopreservation. Blood samples were also obtained from healthy volunteers in Japan. The blood was diluted 2:1 (blood:RPMI-1640) and then PBMCs were purified by a density gradient centrifugation using Ficoll-Paque Plus (GE Healthcare Life Sciences, Cat# 17-1440-03) and stored in liquid nitrogen until further use. Preliminary comparisons indicated that fresh PBMCs from UK donors and cryopreserved PBMCs from donors in Japan showed similar Vγ9Vδ2 T-cell induction profiles under matched culture conditions.

### Cell culture

Cells were cultured at 37 °C and 5% CO_2_ and were tested once a month for mycoplasma using a MycoAlert^®^ mycoplasma detection kit according to the manufacturer’s instructions (Lonza, Basel, Switzerland). Cell lines were validated based on morphology, characteristics, and phenotypic markers according to the American Type Culture Collection (ATCC) or the German Collection of Microorganisms and Cell Cultures (Leibniz Institute DSMZ).

### Cancer cell lines

The following cell lines were used (reference for cell line information included in brackets): lung carcinoma A549 (ATCC^®^ CCL-185™), Chronic Myelogenous Leukaemia K562 (ATCC^®^ CRL-3344™), Acute Myeloid Leukaemia THP-1 (TIB-202), and pancreatic adenocarcinoma DAN-G (DSMZ ACC249). K562 cells were grown in R10 as suspension cultures and split 1:10 once or twice a week. A549 (R10 media) and DAN-G (R10 media) cell lines are adherent, and were passaged by detachment with D-PBS + 2 mM EDTA and split 1:10-1:20 once a week. A549 and THP-1 were engineered for the study as described below, to remove expression of butyrophilin 3A family members.

### T-cell lines

PBMCs were primed with CBM588 tablet or spores (Miyarisan Pharmaceutical Co. Ltd. Tokyo, Japan), or (*E*)-1-Hydroxy-2-methyl-2-butenyl 4-pyrophosphate lithium salt (HMBPP) (Merck) at concentrations indicated and cultured in priming media (RPMI-1640, 100 U/mL Penicillin, 100 µg/mL Streptomycin, 2 mM L-Glutamine, 1X non-essential amino acids, 1 mM sodium pyruvate, 10 mM HEPES buffer, 10% FBS (Gibco, MA, USA) and 20 IU/mL IL-2 (Aldeslukin, Proleukin; Prometheus, San Diego, CA, USA) for two weeks. For proliferation assays, the PBMCs were pre-labelled with 1 µM CFSE, using a CellTrace™ CFSE Cell Proliferation Kit according to manufacturer’s instructions (ThermoFisher Scientific, Waltham, MA, USA) (Gating strategy in **Supplementary Figure 2**).

### Generating butyrophilin 3A knockout cells

A549 and THP-1 BTN3A knockout cells were generated using a CRISPR/Cas9 lentiviral system based on the lentiCRISPR v2 plasmid (Addgene plasmid #52961, a kind gift from Feng Zhang). The puromycin selection gene was replaced with truncated (t) NGFR (CD271) by the Gibson Assembly technique to aid selection of knockout cells (14). The gRNA (5’-ATCAGCGTCTTCACCCACCA TGG-3’PAM sequence underlined) targeting all three BTN3A isoforms (BTN3A1, A2 and A3) was cloned into the lentiCRISPR v2-tNGFR plasmid as described by Addgene. The knockout plasmid was transformed into XL10 gold competent cells (Agilent, CA, USA) for amplification and plasmid Minipreps performed using PureLink™ Quick Plasmid Kit (Life Technologies, CA, USA). For transfection, the lentiCRISPR v2-BTN3A KO gRNA-tNGFR plasmid, the pMD2.G envelope plasmid (Addgene plasmid #12259) and the psPAX2 packaging plasmid (Addgene plasmid #12260) were combined in Opti-MEM (Gibco) media with the addition of 1µg/µL polyethylenimine (Merck). These were incubated at room temperature for 15 min then added to HEK293T cells that were cultured in D10 media (as for R10, but with DMEM (Merck)) and incubated at 37 °C with 5% CO_2_ for three days, with media being removed and replaced with fresh D10 each day. The supernatant media from 48 and 72 hours post-transfection were kept, combined, centrifuged at 800 x g for 5 min and filtered using a 0.45 µm filter (Fisher Scientific, MA, USA). A549 or THP-1 cells were plated at 100,000 cells per 24-well in 1 mL of R10 media and cultured overnight. The next day media was replaced with 500 µL fresh R10 and 500 µL lentivirus supernatant with 5 µg/mL polybrene (Sigma Aldrich, MO, USA) and cells were centrifuged at 400 x g for 2 h for spinfection. Cells were cultured for 7 days then tested for BTN3A knockout compared to A549 or THP-1 wild-type cell lines by antibody staining towards co-marker CD271/tNGFR (clone ME20.4-1.H 4, (Miltenyi Biotec, Bergisch Gladbach, Germany) and CD277/BTN3A (clone BT3.1, also known as clone 20.1, Miltenyi Biotec) surface antibodies (CD277 antibody required a 45 min incubation at 4°C; standard 20 min incubation on ice used for all other surface antibodies). Human FcR blocking reagent (Miltenyi Biotec) was used for THP-1 cells. The A549 or THP-1 BTN3A knockout cell lines were cloned by limiting dilution and clones were confirmed for knockout by surface antibody staining, genomic sequencing to reveal insertions or deletions, and functional testing with Vγ9Vδ2 T-cell lines following pretreatment with 50 µM Zoledronate (Sigma Aldrich).

### *In vitro* induction of CBM588 or HMBPP-reactive T-cells from PBMCs

A suspension of CBM588 tablets was made by dissolving the tablets in PBS (100 mg/mL) and vortexing it for 1 min. Human PBMCs from healthy donors were incubated with 2.5, 5 or 10 mg/mL of CBM588 suspension or 1, 10 or 100 ng/mL of HMBPP in 96U wells (1 x10^6^ PBMCs per well) with 200 µL per well of priming media for 12 days, with the 50% of the media being changed two times per week. Post optimisation, the standard concentrations of CBM588 tablet or HMBPP used for priming T-cells was 10 mg/mL and 10 ng/mL respectively. Purified *Clostridium butyricum* spores were used alongside tablet suspensions to confirm that observed effects were attributable to the bacterial spores rather than tablet excipients.

### Phenotyping of PBMCs primed with CBM588 or HMBPP

*In vitro* primed T-cells were washed and stained with Fixable Live/Dead Violet Dye (ViViD, ThermoFisher) (1/40 pre-dilution then 1/50 for staining), and surface stained with following antibodies: For the antibody panel used in the UK, CD4 BV510 (SK3, 1/100 dilution: BioLegend, San Diego, CA, USA), Vδ2 PE (REA771, 1/100 dilution; Miltenyi Biotec), CD3 PerCP (UCHT1, 1/100 dilution; BioLegend), γδ TCR APC (REA591, 1/100 dilution; Miltenyi Biotec), Vγ9 APC-Vio770 (REA470, 1/100 dilution; Miltenyi Biotec), CD8 PE-Vio770 (REA734, 1/200 dilution; Miltenyi Biotec), αβ TCR FITC (IP26, 1/100 dilution; BioLegend). For the panel used in Japan, Vδ2 VioBlue (REA771, 1/100 dilution; Miltenyi Biotec), Vδ1 PE-eFluor610 (TS8.2, 1/25 dilution; eBioscience, San Diego, CA, USA), Vγ9 TCR APC-Vio770 (REA771, 1/100 dilution; Miltenyi Biotec), αβ TCR PerCP/Cy5.5 (IP26, 1/100 dilution; BioLegend), γδ TCR FITC (REA591, 1/100 dilution; Miltenyi Biotec), CD3 BV785 (UCHT1, 1/50 dilution; BioLegend), CD8 BV570 (RPA-T8, 1/100 dilution; BioLegend), CD4 BV750 (SK3, 1/100 dilution; BioLegend), CD69 BV421 (FN50, 1/100 dilution; BioLegend), PD-1 APC (MIH4, 1/50 dilution; BioLegend), CD137 BV711 (4B4-1, 1/50 dilution; BioLegend), CD103 PerCP-eFluor710 (Ber-ACT8, 1/25 dilution; eBioscience), CD38 AF647 (HIT2, 1/100 dilution; BioLegend), CD39 BV605 (A1, 1/50 dilution; BioLegend), CD56 BV510 (HCD56, 1/50 dilution; BioLegend), TIM3 PE (F38-2E2, 1/25 dilution; BioLegend), 7AAD (1/50 dilution; BioLegend), After incubation for 20 min on ice, the cells were fixed with 2% paraformaldehyde made in-house using formaldehyde or purchased commercially (Nacalai Tesque, Kyoto, Japan, Cat# 09154-85), and the levels of protein surface expression were analyzed by flow cytometry using a FACS Canto II (BD Biosciences, New Jersey, USA) or Cytek Northern Lights (Cytek Japan). The data obtained by flow cytometry were analyzed with FACS Diva v9.0 (BD) and FlowJo software v10 (Tree Star). Gating strategies shown in **Supplementary Figures 3, 4**.

### T107 T-cell activation assay

Unprimed, CBM588- and HMBPP-primed T-cells were rested in R5 (as for R10 with 5% FBS) for 24 h before assay. Typically, 1×10^5^ T-cells and 2×10^5^ target cells, 5 mg/mL CBM588 (per well), 1 μM HMBPP (262 ng/mL) or 2 mL of CD3-CD28 Dynabeads™ (ThermoFisher) were co-incubated for 4 h with 30 μM TAPI-0 (cat# sc-203410, Santa Cruz Biotechnology, Texas, USA), and antibodies directed against TNF (cA2, PE-Vio770, 1/100 dilution; Miltenyi Biotec) and CD107a (H4A3, FITC, 1/100 dilution; BD) or (H4A3, BV421, 1/100 dilution; BioLegend), were also included at the start of the assay (15). Following incubation, cells were washed and stained with ViViD, and the following conjugated antibodies: For antibody panel used in the UK, Vδ2 PE (REA771, 1/100 dilution; Miltenyi Biotec), CD3 PerCP (UCHT1, 1/100 dilution; BioLegend), αβ TCR BV510 (IP26, 1/100 dilution; BioLegend), γδ TCR APC (REA591, 1/100 dilution; Miltenyi Biotec), Vγ9 TCR APC-Vio770 (REA771, 1/100 dilution; Miltenyi Biotec). For the panel used in Japan, Vδ2 PE (REA771, 1/100 dilution; Miltenyi Biotec), CD3 BV785 (UCHT1, 1/50 dilution; BioLegend), αβ TCR PerCP/Cy5.5 (IP26, 1/100 dilution; BioLegend), γδ TCR FITC (REA591, 1/100 dilution; Miltenyi Biotec), CD8 BV570 (RPA-T8, 1/100 dilution; BioLegend), CD4 BV750 (SK3, 1/100 dilution; BioLegend) and levels of protein expression were analyzed by flow cytometry using a FACS Canto II (BD Biosciences) followed by analysis using FACS Diva v9.0 (BD) or Cytek Northern Lights (Cytek Japan) and FlowJo v10 software (Tree Star). Gating strategy in **Supplementary Figure 5A**.

### Flow cytometry-based cancer cell killing assays

1×10^5^ target cell lines were plated in 96U-well plates, and CBM588-primed T-cells added to give the desired T-cell to target cell line ratio. Cells were co-cultured in 200 μL of target-cell media supplemented with 20 IU of IL-2 and 25 ng/mL of IL-15 and incubated for 72 h. Before collection, 1×10^5^ A549-GFP cells were added to each well to allow the number of target cells that remained in experimental and control wells to be quantified. Cells were washed three times with chilled D-PBS EDTA (2 mM) then stained in the assay plates with ViViD then CD3 APC (clone UCHT1, BioLegend) to allow dead cells and T-cells to be gated out, leaving viable target cells for analyses (**Supplementary Figure 5B**). The data were analyzed by flow cytometry using a FACS Canto II (BD Biosciences) followed by analysis using FACS Diva v9.0 (BD) or Cytek Northern Lights (Cytek Japan) and FlowJo v10 software (Tree Star). Percentage killing was calculated as previously described (16).

### Statistics and reproducibility

Data and statistical analysis were performed using Prism 10(GraphPad Software). Statistical tests applied as indicated in the legends: Two-tailed Wilcoxon matched-pairs signed rank test, Fisher’s exact test, Two-tailed Mann–Whitney test, Log-rank test and Kaplan–Meier survival analysis. Raw data values can be found in the **Supplementary Excel File**.

## Results

### The probiotic CBM588 induces Vγ9Vδ2 T-cells from healthy PBMC *in vitro*

**Figure 1:**
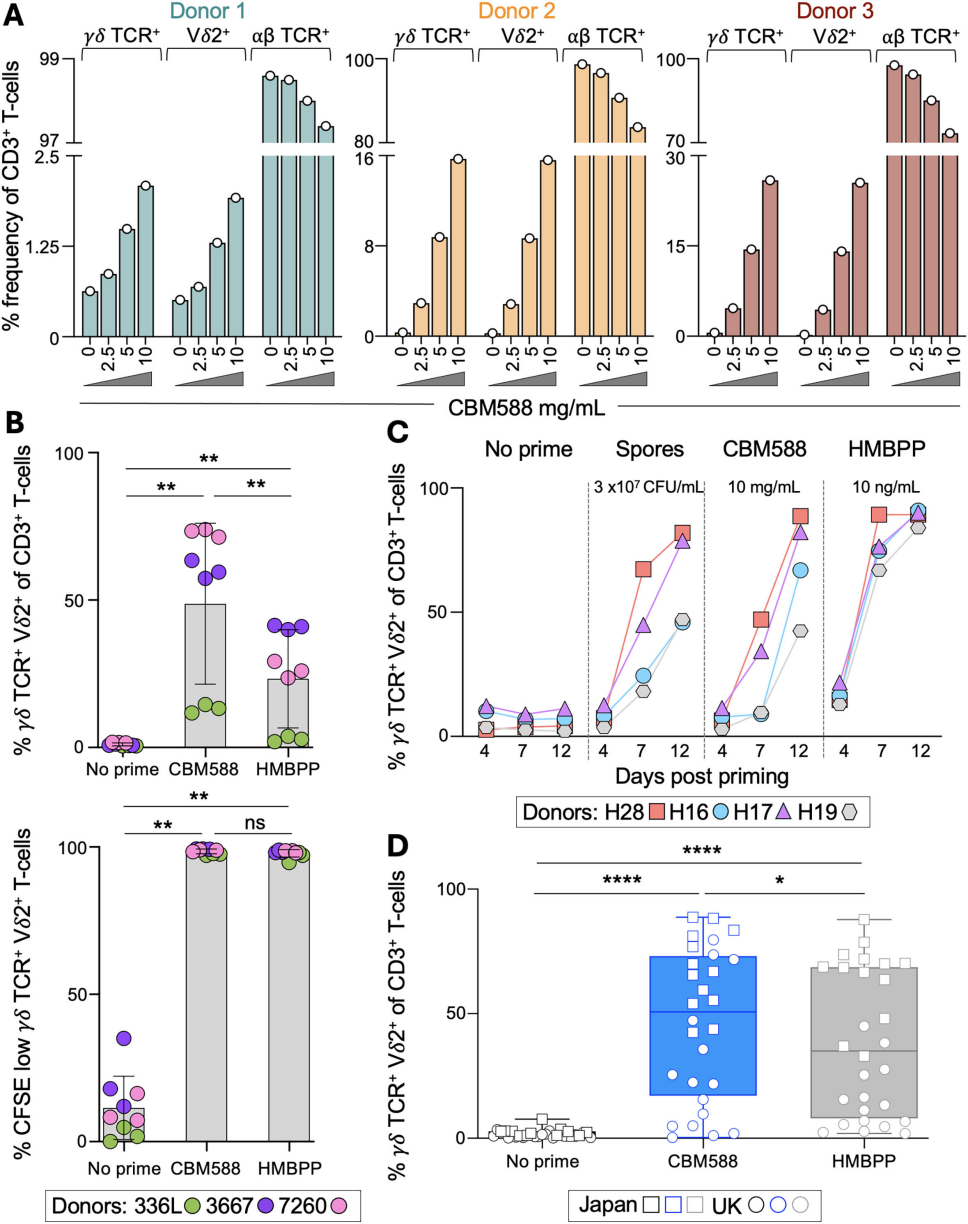
*In vitro* induction of γδ TCR^+^ T-cells by CBM588 tablet or bacterium spores in healthy donors. **(A)** PBMCs from healthy donors were stimulated for 12 days with a suspension of CBM588 at the concentrations shown. Flow cytometry gating strategies, αβ TCR^+^ T-cell subset definitions, and co-receptor and Vγ9 expression within the Vδ2^+^ T-cell population are shown in **Supplementary Figure 6**. **(B)** Upper: γδ TCR^+^ Vδ2^+^ frequency among CD3^+^ T-cells after priming of PBMCs for 14 days from three donors with CBM588 (10 mg/mL) or HMBPP (10 ng/mL). Performed as triplicate priming conditions for each donor according to the key. Error bars depict standard deviation. Two-tailed Wilcoxon matched-pairs signed-rank test (**p = 0.0039). Lower: the same conditions and donors as before, gating on CFSE low γδ TCR^+^ Vδ2^+^ T-cells as an indicator of proliferation. Error bars depict standard deviation. Two-tailed Wilcoxon matched-pairs signed-rank test (**p = 0.0039). **(C)** Frequency of γδ TCR^+^ Vδ2^+^ T-cells among CD3^+^ T-cells following stimulation of PBMCs from four healthy donors with CBM588 spores (3×10^7^ CFU/mL), CBM588 tablet suspension (10 mg/mL), or HMBPP (10 ng/mL) at days 4, 7 and 12 post stimulation. Results at other concentrations of spore, CBM588 tablet and HMBPP are shown in **Supplementary Figure 7**, alongside αβ TCR^+^ and γδ TCR^+^ subset definitions. **(D)** Frequency of γδ TCR^+^ Vδ2^+^ T-cells among CD3^+^ T-cells following 12 days of stimulation with CBM588 (10 mg/mL) or HMBPP (10 ng/mL) in healthy donors (n = 28). Squares indicate donors in Japan, and circles indicate donors in the United Kingdom. Two-tailed Wilcoxon matched-pairs signed-rank test (****p < 0.0001 and *p = 0.0294). Box plot showing the median, and whiskers at minimum and maximum values.

We hypothesized that CBM588 may exert its striking clinical benefit for NSCLC (9, 10) by inducing T-cells capable of recognizing cancer cells. To test this, we stimulated PBMCs from healthy donors with a suspension of CBM588 tablets for 7 and 12 days, as described in the Materials and Methods. Flow cytometry analysis revealed that CBM588 induced a dose-dependent increase in γδ TCR^+^ T-cells, accompanied by a relative decrease in αβ TCR^+^ T-cells within the CD3^+^ population (**Figure 1A**). The frequency of γδ TCR^+^ T-cells closely matched that of Vδ2^+^ T-cells, suggesting that CBM588 preferentially expanded the Vδ2^+^ subset without broadly increasing other γδ T-cell populations (**Figure 1A**). Further staining confirmed that the Vδ2^+^ T-cells co-expressed Vγ9, identifying them as Vγ9Vδ2 T-cells (**Supplementary Figure 6A**). Analysis of αβ TCR^+^ T-cells showed a slight shift in co-receptor expression, with a subtle decrease in CD4^+^ and increase in CD8^+^ T-cells, while the proportions of double-positive and double-negative subsets remained unchanged (**Supplementary Figure 6B**). Among the CBM588-induced Vδ2^+^ T-cells, the majority were CD4 and CD8 double negative, with approximately 15% expressing CD8 (**Supplementary Figure 6C**).

We labelled the PBMCs from three additional healthy donors with CFSE to assess T-cell proliferation following priming with CBM588. As a positive control, we included (*E*)-4-Hydroxy-3-methyl-but-2-enyl pyrophosphate (HMBPP), a microbial metabolite from the non-mevalonate isoprenoid biosynthesis pathway (17). HMBPP activates Vγ9Vδ2 T-cells by acting as a molecular ‘glue’ that promotes the heteromeric association of butyrophilins BTN3A1 and BTN2A1, forming a TCR ligand (18, 19). Consistent with observations from the first three donors, exposure to CBM588 led to a significant increase in the proportion of γδTCR^+^Vδ2^+^ T-cells (p = 0.0039), which in most donors exceeded the response observed with HMBPP (**Figure 1B**). In both the CBM588 and HMBPP conditions, more than 97% of the γδTCR^+^Vδ2^+^ T-cells were within the CFSE low population, indicating proliferation, compared to the CFSE high cells present in the unprimed control (**Figure 1B**). Collectively, these data demonstrate that Vδ2^+^ T-cells are actively expanding in response to CBM588 stimulation, rather than simply reflecting a relative decline in the αβ TCR^+^ T-cell population.

Encouraged by these findings, we tested PBMCs from four additional healthy donors to confirm that the observed expansion of Vδ2^+^ T-cells was attributable to *Clostridium butyricum* spores rather than tablet excipients. Robust induction of Vδ2^+^ T-cells was observed following stimulation with either the CBM588 tablet suspension, the purified spore preparation, or HMBPP (**Figure 1C**, **Supplementary Figure 7A**), with no expansion of other T-cell subsets (**Supplementary Figure 7B**). This confirmed that the expansion was driven by bacterial spores and not by components of the tablet formulation.

Combined priming experiments using PBMCs from 28 donors in the UK and Japan confirmed that CBM588 tablet suspension significantly expanded Vδ2^+^ T-cells after 12 days, increasing the median frequency from 1.31% in unprimed cultures to 50.7% following stimulation (p < 0.0001; **Figure 1D**). Notably, Vδ2^+^ T-cell frequencies were generally higher in the donors from Japan, with ~30% of UK donors showing no expansion. This difference may reflect methodological or population-specific factors, or the fact that CBM588 is widely used in Japan to treat diarrhoea (20), potentially stimulating T-cell memory responses. Further investigation will be required to clarify this observation. These experiments also showed that fresh PBMCs from UK donors and cryopreserved PBMCs from Japanese donors behaved comparably under matched conditions. Together, these data indicate that CBM588-derived bacterial components can expand Vγ9Vδ2^+^ T-cells from healthy donor PBMCs. As some Vγ9Vδ2^+^ T-cells are known to kill cancer cells in response to tumour-derived pyrophosphates (21–24), we next investigated whether CBM588-induced T-cells could respond to cancer cells.

### CBM588-primed γδ T-cells recognize cancer cells via BTN3A

**Figure 2:**
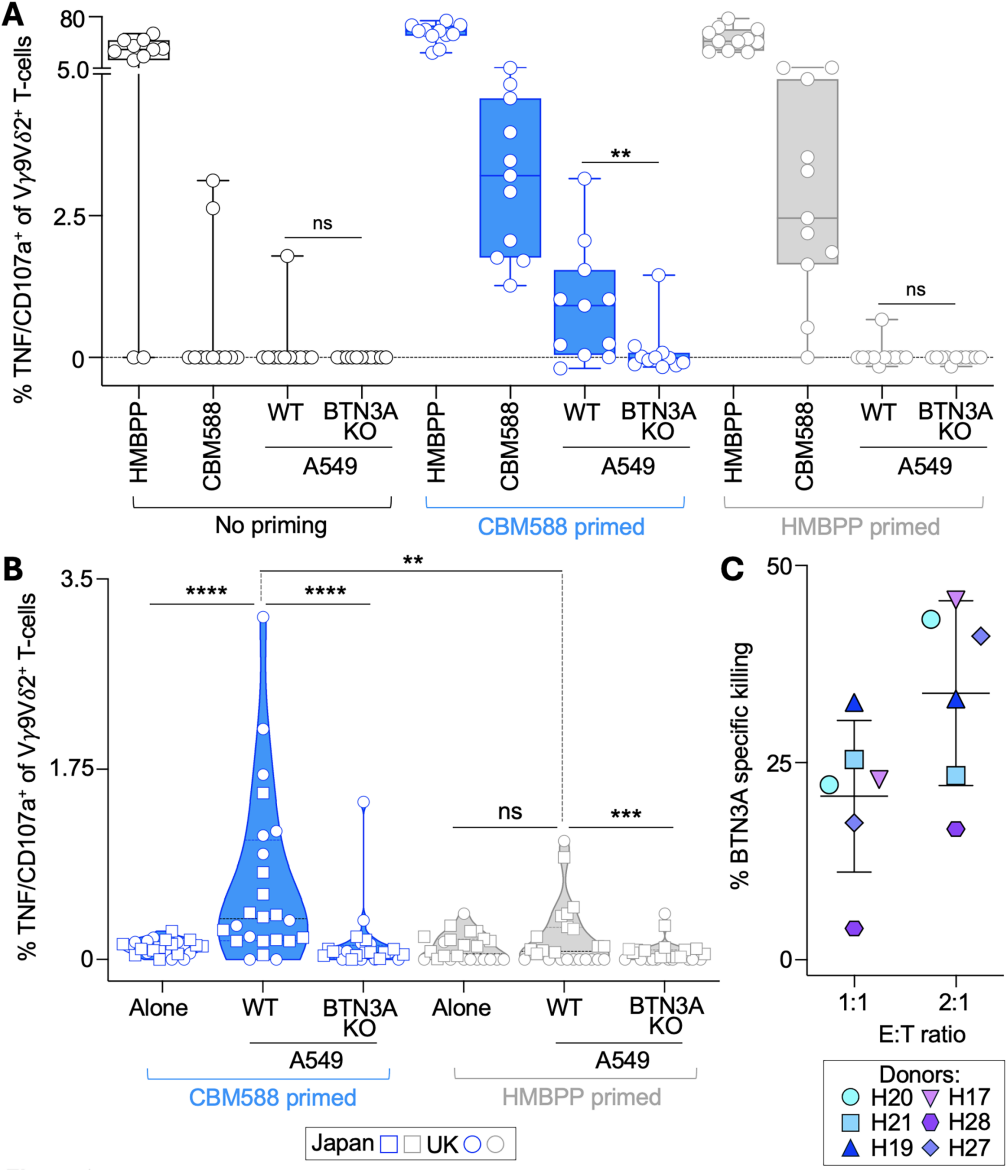
Cancer reactivity of CBM588 tablet-primed Vγ9Vδ2 T-cells from healthy donors. **(A)** PBMCs from 11 healthy donors in the United Kingdom were left unprimed or primed with CBM588 tablet suspension (10 mg/mL) or HMBPP (10 ng/mL). After 12 days in culture, Vγ9Vδ2^+^ T-cell reactivity was assessed by T107 assay (CD107a/TNF) in response to CBM588 tablet suspension, HMBPP, lung cancer A549 wild-type or BTN3A knockout (KO) cells. Background levels of CD107a/TNF from unstimulated T-cells were subtracted. Data is presented as a box plot with median values and whiskers indicating minimum and maximum values. Two-tailed Wilcoxon matched-pairs signed rank test (**p = 0.0039). **(B)** Vγ9Vδ2^+^ T-cell reactivity (T107 assay) from CBM588- or HMBPP-primed PBMCs from healthy donors in the United Kingdom (same as in a, without background subtraction) and Japan (as indicated in the key) in response to A549 and A459-BTN3A KO cells. Two-tailed Wilcoxon matched-pairs signed rank test (**p = 0.0051; ***p = 0.0002; ****p < 0.0001)). See **Supplementary Figure 8** for αβ TCR^+^ subset reactivity. **(C)** Cytotoxicity assay of CBM588-primed T-cells from six healthy donors against A549 or A549-BTN3A KO cells over 72 hours. Data shows BTN3A-dependent killing, calculated by subtracting the killing of A549-BTN3A KO cells from that of wild-type A549 cells. See **Supplementary Figure 9** for recognition of additional cancer cell types (leukaemia and pancreatic).

To assess whether CBM588-primed γδ T-cells could recognize cancer cells, we used the lung cancer cell line A549 and generated a BTN3A knockout variant (A549-BTN3A KO) by CRISPR/Cas9 disruption of all three BTN3A isoforms, as described in the Materials and Methods. BTN3A1 is required for recognition of phosphoantigens such as HMBPP and isopentenyl pyrophosphate (IPP) by Vγ9Vδ2 T-cells (18), enabling us to test whether recognition was mediated via tumour-derived mevalonate metabolites (22). We assessed T-cell responses by flow cytometry at the single-cell level, measuring CD107a (a surrogate for degranulation and cytotoxic activity (25)) and surface TNF using the T107 assay. In the UK-based cohort, 2 of 11 donors showed baseline reactivity to CBM588, and only one donor exhibited recognition of A549 lung cancer cells without priming. Following CBM588 priming, all 11 donors responded to CBM588, and 7 of 11 recognized A549 lung cancer cells (**Figure 2A**). This recognition was abrogated in A549-BTN3A KO cells, consistent with a BTN3A-dependent mechanism involving IPP accumulation (22). While priming with HMBPP successfully induced CBM588-reactive T-cells, it did not confer cancer cell recognition in any donor except the single individual with pre-existing reactivity, highlighting a key distinction between HMBPP and CBM588 (**Figure 2A**). Both CBM588 and HMBPP-primed T-cells responded robustly to HMBPP, but only CBM588 priming induced cancer-reactive Vγ9Vδ2 T-cells, suggesting that CBM588 drives the expansion of T-cell clonotypes or activation states capable of recognising tumour-derived phosphoantigens. We then extended the analysis to include donors in Japan, combining data across 24 healthy individuals. CBM588-primed T-cells demonstrated a median of 0.38% CD107a^+^/TNF^+^ responses among total CD3^+^ T-cells in response to wild-type A549 cells compared to 0.064% against BTN3A-deficient targets (**Figure 2B**; **** p < 0.0001), confirming robust and reproducible BTN3A-dependent activation. In contrast, HMBPP priming did not increase A549 reactivity above baseline (**Figure 2B**). Importantly, γδ TCR negative T-cells showed no response to A549 lung cancer cells under any priming condition (**Supplementary Figure 8**). CBM588-primed cells also recognised acute myeloid leukaemia cells (THP-1), again in a BTN3A-dependent manner (**Supplementary Figure 9A**). Together, these findings indicate that CBM588 can prime Vγ9Vδ2 T-cells with the capacity to recognise cancer cells through a BTN3A-dependent mechanism. We next asked whether the CBM588-primed T-cells could kill cancer cells.

### CBM588-primed T-cells can kill cancer cell lines in a BTN3A-dependent manner and recognise different cancer types

To confirm the cytotoxic potential of CBM588-primed T-cells, we performed a flow cytometry-based killing assay as previously described (16). In six healthy donors tested, CBM588-primed T-cells demonstrated BTN3A-dependent cytotoxicity against A549 lung cancer cells. At a 1:1 effector-to-target ratio over 72 hours, CBM588 priming resulted in a median of 22.5% BTN3A-specific killing, calculated by subtracting killing of BTN3A-deficient targets from killing of wild-type A549 cells (p < 0.01; **Figure 2C**). This effect was observed consistently across donors and increased further at a 2:1 effector-to-target ratio supporting a dose-dependent cytotoxic response. We next explored whether this effect extended beyond lung cancer. CBM588 exposure enhanced the responsiveness of donor γδ T-cells against additional cancer cell types, including pancreatic adenocarcinoma and leukaemia (**Supplementary Figure 9B**), indicating that CBM588-induced Vγ9Vδ2 T-cells can recognise multiple tumour types. We next investigated the impact of CBM588 on γδ T-cell responses in lung cancer patients receiving immune checkpoint inhibitors (ICIs), with or without concurrent administration of CBM588.

### Vγ9Vδ2 T-cells in lung cancer patients treated with ICIs and CBM588 exhibit increased activation and infiltration into cancerous site

**Figure 3:**
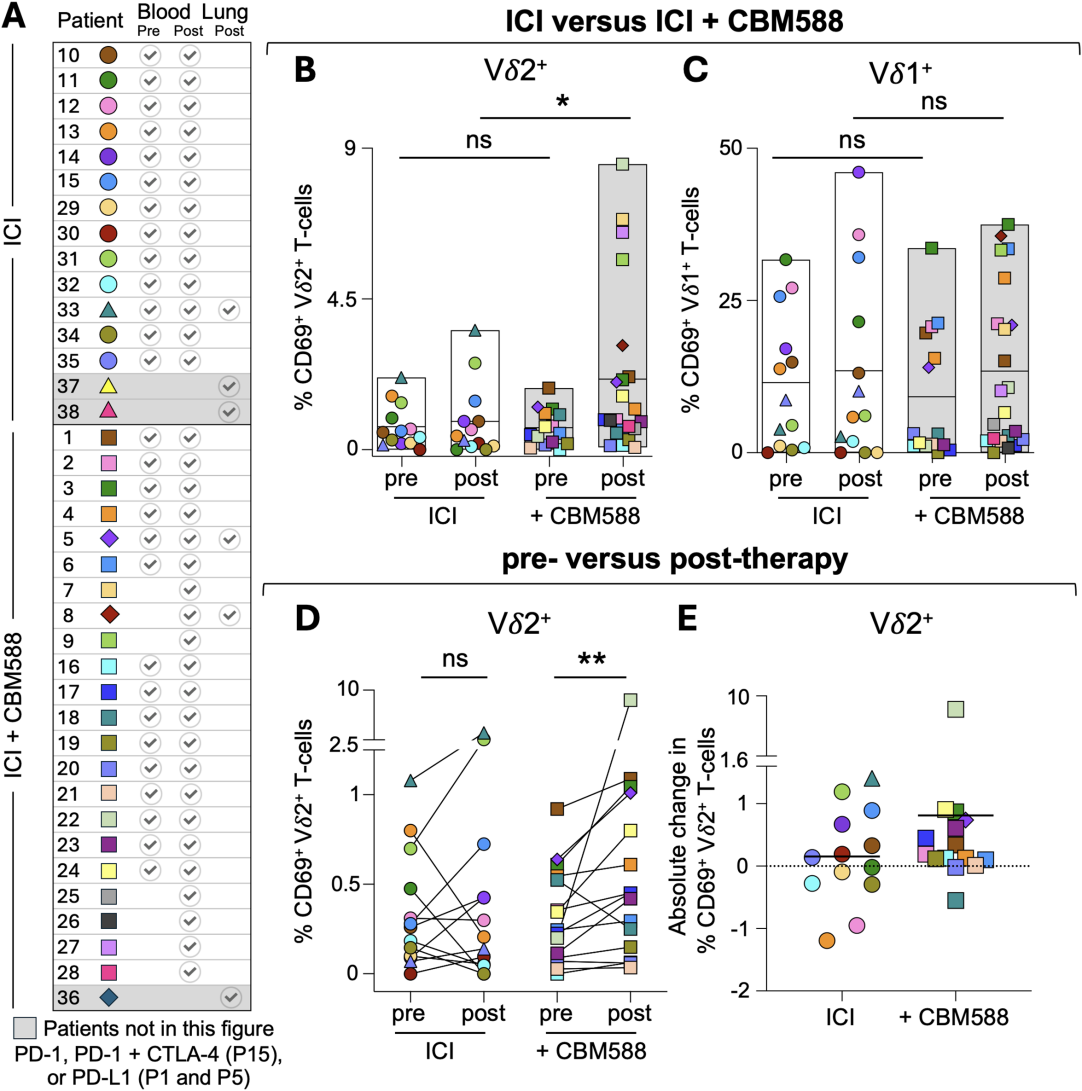
Frequency, activation status and cancer reactivity of Vδ2^+^ T-cells in the peripheral blood of lung cancer patients treated with ICI and CBM588. **(A)** Lung cancer patients (study total = 38) based in Japan received immune checkpoint inhibitor (ICI) therapy with or without CBM588. Each patient is represented by a unique symbol and colour combination. PBMCs were collected pre- and/or post-treatment; pleural effusion samples were collected post-treatment from six patients. See **Supplementary Figure 1** for patient demographics and sampling details. **(B)** Comparison of CD69^+^ Vδ2^+^ T-cells between ICI and ICI+CBM588 treatment groups using all patients with PBMC samples (n = 35). Two-tailed Mann–Whitney test (*p = 0.043). **(C)** Comparison of CD69^+^ Vδ1^+^ T-cells between ICI and ICI+CBM588 treatment groups using all patients with PBMC samples (n = 35). Two-tailed Mann–Whitney test. **(D)** Frequency of CD69^+^ Vδ2^+^ T-cells from patients with available pre- and post-treatment PBMC samples (n = 28). Two-tailed Wilcoxon matched-pairs signed rank test (**p = 0.0041). See **Supplementary Figures 10, 11** for frequency of, and expression of other activation markers, of various T-cell subsets. **(E)** Absolute change in CD69 expression on Vδ2^+^ T-cells post treatment for 28 patients. The median values are shown by the solid line.

**Figure 4:**
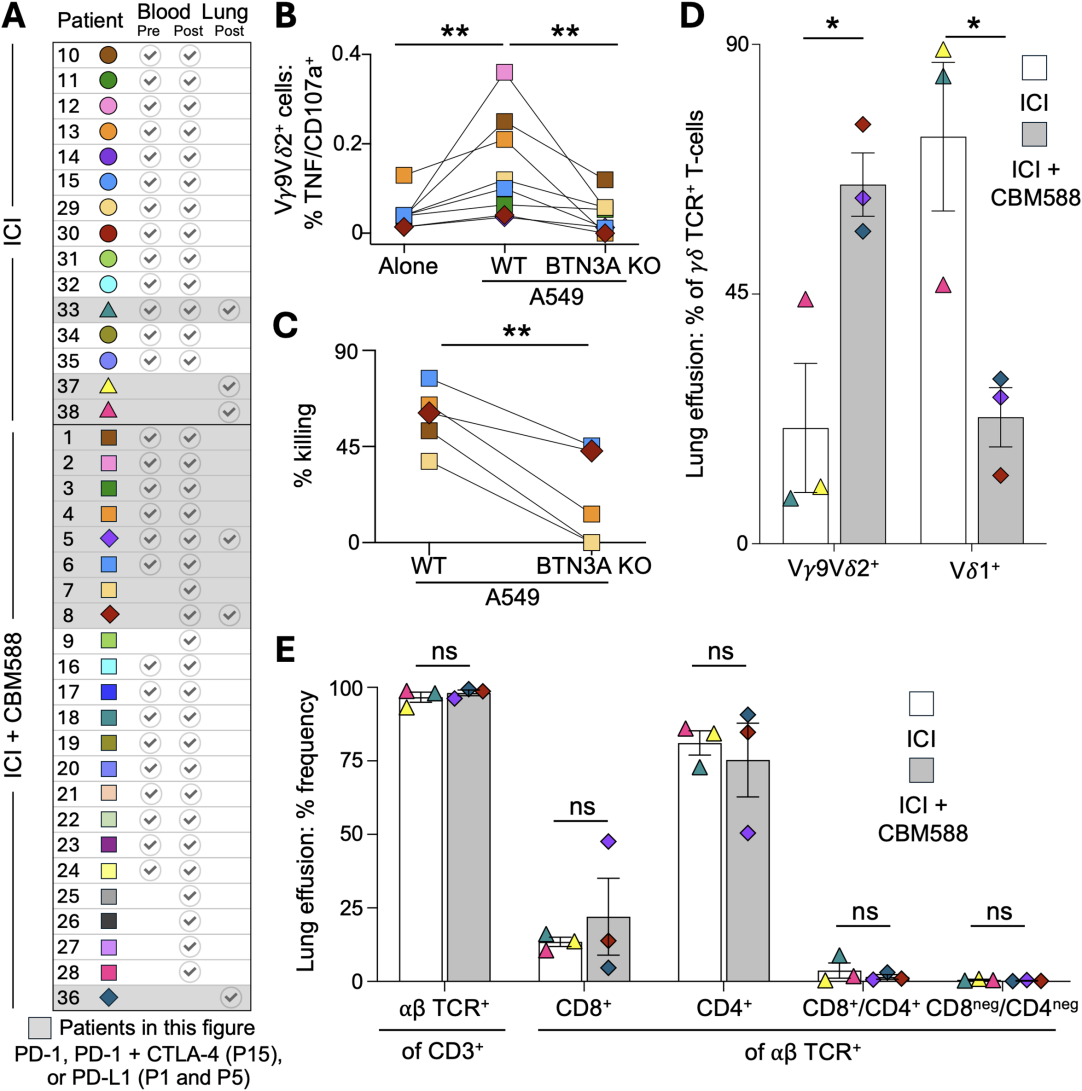
Frequency of Vδ2^+^ T-cells in lung effusions of lung cancer patients treated with ICI and CBM588. **(A)** Lung cancer patients (study total = 38) based in Japan received immune checkpoint inhibitor (ICI) therapy with or without CBM588. Each patient is represented by a unique symbol and colour combination. PBMCs were collected pre- and/or post-treatment; pleural effusion samples were collected post-treatment from six patients. See **Supplementary Figure 1** for patient demographics and sampling details. **(B)** PBMCs from patients treated with ICI+CBM588 were primed *in vitro* with CBM588 tablets and assessed for cancer reactivity using the T107 assay (CD107a/TNF) against A549 and A549-BTN3A KO lung cancer cells. Two-tailed Wilcoxon matched-pairs signed rank test (**p = 0.0078). **(C)** Killing assay using CBM588-primed PBMCs from patients treated with ICI+CBM588, challenged with A549 or A549-BTN3A KO cells (1:1 ratio, 72 h). Paired two-tailed Student’s t-test (**p = 0.0040). **(D)** Proportion of γδ TCR^+^ T-cell subsets in pleural effusions from lung cancer patients treated with ICI with or without CBM588. Error bars represent standard error of the mean. Paired two-tailed Student’s t-test (*p = 0.0276) for Vγ9Vδ2^+^ and (*p = 0.0248) for Vδ1^+^ T cells. **(E)** Proportion of αβ TCR^+^ T-cell subsets in pleural effusions from lung cancer patients treated with ICI with or without CBM588. Error bars represent standard error of the mean. Paired two-tailed Student’s t-test.

To investigate the frequency and activation status of Vγ9Vδ2^+^ T-cells in lung cancer patients undergoing treatment with ICIs, we analysed PBMCs from NSCLC patients who had been enrolled in trials of CBM588 therapy (patient details in **Figure 3A**, **Supplementary Figure 1**). For 28 patients, paired PBMC samples pre- and post-treatment with ICI (n = 13) or ICI+CBM588 (n = 15) were available for comparison, and these showed no change in the proportions of αβ TCR positive or γδ TCR positive subsets following therapy, including Vδ2^+^ T-cells (**Supplementary Figure 10**). Next, we examined various activation markers on peripheral blood T-cells subsets and compared their pre- and post-therapy levels between the ICI (n = 13) and ICI+CBM588 (n = 22) patient cohorts. There was significant increase (p = 0.043) in the proportion of post-therapy CD69^+^Vδ2^+^ T-cells in patients using ICI+CBM588 compared to ICI alone, suggesting greater *in vivo* activation of this subset (**Figure 3B**, **Supplementary Figure 11**). In contrast, Vδ1^+^ T-cells did not show any differences in CD69 expression between the ICI and ICI+CBM588 cohorts (**Figure 3C**, **Supplementary Figure 11**). We also observed a significant increase (p= 0.0041) in the percentage of CD69^+^Vδ2^+^ T-cells between pre- and post-therapy samples for patients taking CBM588, whereas no such increase was seen in the ICI-only cohort (**Figures 3D, E**, **Supplementary Figure 11**). PBMCs from these patients (**Figure 4A**), when primed with CBM588 for 12 days, were capable of recognizing (**Figure 4B**) and killing (**Figure 4C**) A549 lung cancer cells in a BTN3A-dependent manner. We also examined malignant pleural effusions from lung cancer patients treated with ICI alone or in combination with CBM588. The proportion of Vγ9Vδ2 T-cells was significantly higher in patients receiving ICI+CBM588 compared to those on ICI alone (**Figure 4D**). This increase in Vγ9Vδ2 cells was accompanied by a relative decrease in Vδ1^+^ T-cells (**Figure 4D**), and no αβ TCR^+^ subsets were altered (**Figure 4E**), indicating that CBM588 changes the balance of γδ T-cell subsets at tumour-associated sites. Overall, these data demonstrate increased activation and intratumoral infiltration of Vγ9Vδ2 T-cells in lung cancer patients treated with CBM588, providing a plausible mechanistic basis for the improved patient outcomes observed across multiple clinical studies (9–12).

### Activation of Vδ2^+^ T-cells in lung cancer patients correlates with improved outcomes

**Figure 5:**
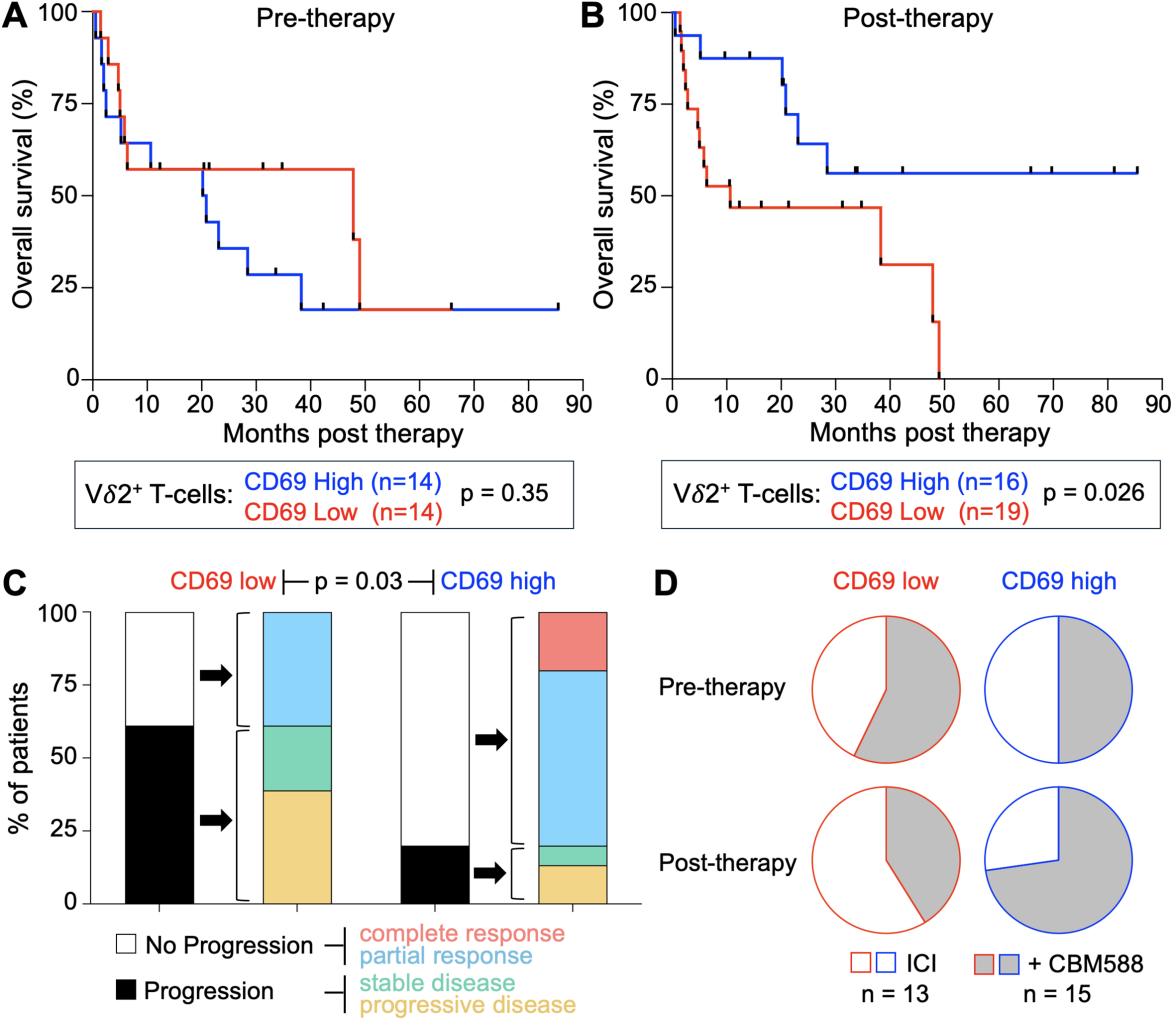
High CD69 expression on Vδ2^+^ T-cells correlates with improved outcomes in lung cancer patients receiving immunotherapy. **(A)** Kaplan–Meier analysis of overall survival in lung cancer patients (n = 28, with pre- and post-therapy samples) treated with ICI or ICI+CBM588 (analysed as a combined cohort). Patients were stratified into high or low CD69 expression groups based on CD69^+^Vδ2^+^ T-cell frequency in pre-treatment blood samples, relative to the cohort median. *p* value from a log-rank test. Median values of CD69 are shown in **Supplementary Figure 12**. **(B)** Kaplan–Meier analysis as in **(A)**, based on CD69^+^Vδ2^+^ T-cell frequency in post-treatment blood samples from patients (n = 35, with post-therapy samples). *p* value from a log-rank test. **(C)** Clinical outcomes of patients (n = 33, clinical response category nonevaluable for two patients) stratified by high or low CD69 expression on Vδ2^+^ T-cells post-therapy. Outcomes categorized according to the key. Statistical analysis by a Fisher’s exact test. **(D)** Distribution of patients (n = 28, with pre- and post-therapy samples) treated with ICI or ICI+CBM588 across the pre- and post-treatment CD69 high/low Vδ2^+^ T-cell categories. See **Supplementary Figure 13** for related analyses based on post-therapy increases or decreases in CD69 expression.

We next assessed the relationship between Vδ2^+^ T-cell activation and clinical outcomes. We combined the ICI and ICI+CBM588 cohorts for this analysis to evaluate whether Vδ2^+^ T-cell activation was associated with survival across the treated population as a whole, independent of treatment allocation. Because the survival benefit of CBM588 has previously been reported (9, 10) analysing treatment arms separately risked conflating treatment effect with biomarker association. The median frequency of CD69 expression on Vδ2^+^ T-cells was calculated, and patients were stratified into two groups: those with CD69 expression above the median and those with expression equal to or below it, both pre- and post-treatment (**Supplementary Figure 12**). CD69 expression before treatment did not correlate with overall survival, but post-treatment expression was strongly predictive (p < 0.03) (**Figures 5A,B**). All patients in the CD69 low group (n = 19) succumbed to disease, whereas 10 of 16 patients in the CD69 high group remained alive at the last follow-up (log-rank p = 0.026), which ranged from 0.044 to 7.12 years (mean 1.68 years) post treatment (**Figure 5B**). Most of the long-term survivors had stable disease, and all three patients who achieved complete remission were in the CD69 high group (**Figure 5C**) and had received CBM588. Fewer than half of patients were CD69 high before treatment with ICI+CBM588; this proportion rose to nearly three-quarters post-treatment (**Figure 5D**). Significantly improved survival and clinical outcomes were also observed when Vδ2^+^ T-cell analysis was based on an increase in CD69 expression following initiation of therapy (**Supplementary Figure 13**).

## Discussion

Here, we demonstrate that the probiotic *Clostridium butyricum* MIYAIRI 588 can expand cancer-reactive Vγ9Vδ2 T-cells *in vitro*, enhance BTN3A-dependent tumour recognition, and is associated in patients with increased peripheral activation and tumour-site enrichment of this subset. Importantly, post-treatment activation of Vδ2^+^ T-cells correlated strongly with improved survival in lung cancer patients, identifying this population as both a potential mechanistic mediator and a candidate prognostic biomarker in the context of probiotic-augmented immunotherapy.

Recent findings suggest that the gut microbiome plays a key role in cancer prognosis and response to treatment. Gajewski and colleagues showed that melanoma growth varied markedly in mice populated with distinct commensal microbiota, with *Bifidobacterium* associated with antitumour effects (7). Oral administration of *Bifidobacterium* was as effective as checkpoint blockade in these animals, with combination treatment almost eliminating tumour outgrowth (7). In humans, the composition of the gut microbiome differs significantly between responders and non-responders to anti-PD1 immunotherapy in metastatic melanoma (5, 6). Clinical trials have also shown that faecal microbiota transplantation can influence the response in patients with anti-PD1-refractory melanoma (26–28). A further study linked specific microbial species in the baseline stool microbiome to improved response rates and prolonged progression-free survival in patients with epithelial tumours (4). Several recent studies have shown that gut microbiome composition serves as a predictive biomarker for response to CD19-targeted CAR-T cell therapy (29, 30), with one particularly striking report demonstrating that supplementation with the newly classified bacterial species *Akkermansia massiliensis* enhances the therapeutic potency of CAR-T cells against B-cell malignancies (31). These findings across multiple malignancies have generated considerable interest in whether probiotics could be harnessed to improve treatment outcomes and cancer survival.

Optimal exploitation of the intestinal microflora in cancer therapy will require a mechanistic understanding of how gut microorganisms exert effects at distant tumour sites. Several possibilities have been proposed, including direct or indirect modulation of the tumour-resident microbiome, alteration of the immune microenvironment, and the systemic effects of microbial metabolites on immune and/or tumour cells. Notably, human unconventional T-cells, including γδ T-cells, can respond to intermediates in microbial metabolic pathways such as those for vitamin B and isoprenoid biosynthesis (22, 32), providing a direct route by which the microbiota can influence systemic T-cell activity. We were interested in whether a probiotic species associated with improved cancer outcomes might be capable of directly priming T-cells that recognize and respond to cancer cells.

Several recent studies have shown that daily administration of CBM588 significantly improves response rates and overall survival in patients with NSCLC and mRCC when used alongside ICIs (9–12). The mechanisms underlying these clinical benefits remain unclear, and only a subset of patients appear to derive full advantage from treatment. In the study by Dizman and colleagues (11), wide differences in metabolic pathways emerged between CBM588-treated and untreated patients over a 12-week period. Here, we show that *in vitro* stimulation of PBMC with CBM588 can stimulate the expansion of a population of cancer-reactive Vγ9Vδ2 T-cells with the capacity to kill cancer cell lines via a BTN3A-dependent mechanism. However, there was substantial variability between donors. Nearly a third of healthy donors showed little or no expansion of Vγ9Vδ2 T-cells in response to CBM588, and in some cases, expanded cells lacked reactivity against cancer cell lines. This variability suggests important differences in TCR specificity or sensitivity to antigen, and further work will be needed to understand how this might relate to differential patient responses to probiotic therapy. Importantly, using PBMCs from lung cancer patients in recent studies conducted in Kumamoto (9, 10), we found that the frequency of activated (CD69^+^) Vδ2^+^ T-cells in peripheral blood increased following CBM588 treatment. In patients receiving ICI+CBM588, the proportion of CD69^+^Vδ2^+^ T-cells was significantly higher than in those receiving ICI alone, suggesting *in vivo* activation of these cells. Notably, elevated levels of CD69 expression on in peripheral blood Vδ2^+^ T-cells post-therapy correlated with improved clinical outcomes and overall survival in lung cancer patients. CBM588 also influenced the γδ T-cell landscape within diseased tissue; lung effusions from patients receiving ICI+CBM588 contained a significantly greater proportion of Vγ9Vδ2 T-cells compared to patients on ICI alone. These findings raise the possibility that CBM588 acts to promote the expansion and activation of cancer-reactive Vγ9Vδ2 T-cells at the cancer site in humans.

Overall, our results support a model in which certain gut-associated bacteria, such as *Clostridium butyricum*, may influence antitumour immune responses through selective effects on the γδ T-cell compartment. These findings open new avenues for understanding and harnessing microbial control of cancer immunosurveillance. In particular, microbiota-derived isoprenoids warrant further investigation. Isoprenoids and their derivatives constitute the largest family of natural organic compounds, with over 65,000 members found across all domains of life (33, 34). Two evolutionarily distinct biosynthetic pathways exist for their production (35), and organisms may employ either or both. The diversity of enzymes and intermediates, especially in the early steps, continues to expand (36). Mammalian cells utilize the mevalonate (MVA) pathway, which produces isopentenyl pyrophosphate (IPP), a known ligand for Vγ9Vδ2 T-cells (22). Most bacteria, by contrast, use the non-mevalonate (MEP) pathway, which generates ligands such as HMBPP that are vastly more potent than IPP in stimulating these cells. Remarkably, IPP and HMBPP differ by only a single hydroxyl group, yet HMBPP is ~10,000-fold more effective at activating cognate T-cells (37).

Our findings demonstrate that CBM588 can directly induce cancer-reactive Vγ9Vδ2 T-cells and remodel the γδ T-cell landscape in both peripheral blood and the tumour site, providing a plausible immunological mechanism for its clinical benefit. We also identify Vδ2^+^CD69^+^ T-cells in patient blood as a robust prognostic marker in NSCLC. These observations are consistent with a comprehensive pan-cancer analysis of over 18,000 human tumours, which identified γδ T-cell infiltration as the strongest predictor of favourable prognosis across 25 malignancies, exceeding the prognostic value of αβ T-cell subsets (38). Future studies should aim to define more precisely the relationship between microbiome modulation and the Vγ9Vδ2 T-cell response in patients. Correlating longitudinal changes in stool microbiome composition with peripheral Vγ9Vδ2 T-cell frequency, activation status, and TCR repertoire dynamics will help determine whether specific microbial signatures are associated with expansion of tumour-reactive clonotypes. High-resolution TCR sequencing before and after CBM588 administration may reveal whether probiotic exposure selectively expands pre-existing tumour-reactive clones or reshapes the repertoire more broadly. In addition, it will be important to determine whether CBM588-induced Vγ9Vδ2 T-cell activation occurs across multiple tumour types and clinical contexts, including settings in which immune checkpoint blockade is not administered. Such studies will help clarify whether the mechanism described here represents a broader tumour-agnostic pathway of microbiome-driven antitumour immunity. Taken together, these results reveal the gut microbiome as a key modulator of systemic antitumour immunity and suggest that its rational manipulation could reshape the immunological landscape of cancer. Probiotic interventions, such as CBM588, may ultimately offer a tractable route to enhance therapeutic efficacy and improve clinical outcomes across diverse malignancies.

### Study limitations

CBM588 has been shown to improve clinical outcomes in NSCLC and RCC, but the mechanisms underlying these effects have remained unclear. In this study, we provide evidence that CBM588 can expand cancer-reactive Vδ2^+^ T-cells from the PBMC of both healthy donors and patients with lung cancer. In patients initiating immune checkpoint blockade alongside CBM588, we also observe marked shifts in the peripheral and tumour-associated γδ T-cell compartment, as well as a correlation between Vδ2^+^ T-cell activation and improved survival. However, several limitations should be acknowledged.

Our study is observational and relies on a relatively modest sample size derived from retrospective clinical cohorts, which limits statistical power and the ability to draw definitive causal inferences. While our data suggest that CBM588 supplementation modulates the Vδ2^+^ T-cell compartment *in vivo*, we cannot rule out the contribution of other factors associated with treatment or patient heterogeneity. In addition, because survival analyses were performed across combined treatment arms, the influence of treatment allocation on outcome cannot be fully separated from the association with CD69 expression, and these findings should therefore be interpreted as exploratory. Although Vδ2^+^ T-cell activation correlated with improved outcomes in our patient cohort, this association does not prove mechanistic linkage, and it remains possible that Vδ2^+^ activation reflects an indirect consequence of improved clinical outcome rather than a driver of therapeutic benefit.

A further limitation relates to the absence of *in vivo* modelling. Human Vγ9Vδ2 T-cells are largely restricted to primates and a small number of other species such as camelids. Rodents, including mice, do not possess a peripheral Vγ9Vδ2 T-cell compartment, nor has a rodent T-cell subset been described that recognises bacterial isoprenoid metabolites via a butyrophilin-dependent system. Conventional murine tumour models are therefore not biologically suited to interrogate this pathway. The most appropriate *in vivo* approach would require non-human primate models, which present substantial ethical and regulatory constraints. As a result, direct *in vivo* mechanistic validation of this specific pathway remains challenging.

CBM588 is also known to induce broad changes in the gut microbiome. As such, the immunological effects observed in patients could result not only from direct action of *C. butyricum*, but also from secondary shifts in microbiota composition, either through expansion of other beneficial species or suppression of inhibitory taxa. Notably, recent studies have shown that certain commensal bacteria can augment checkpoint blockade by driving the migration of activated dendritic cells from the gut to the tumour site, thereby enhancing CD8^+^ T-cell priming and infiltration (39), and that higher systemic levels of microbiota-derived butyrate are associated with improved responses to CAR-T therapy in non-Hodgkin lymphoma (40). These observations highlight the likelihood that multiple immunological mechanisms may operate in parallel to mediate microbiome-driven cancer control. Prospective studies will be essential to validate these associations, clarify causality, and establish whether Vδ2^+^ T-cells contribute directly to improved tumour control in patients receiving CBM588. Our results provide a plausible immunological mechanism for the clinical effects of CBM588 and offer a new framework for exploring microbiome-immune system interactions in cancer.

## Data Availability

The original contributions presented in the study are included in the article/**Supplementary Material**. Further inquiries can be directed to the corresponding authors.
