# Supplementary material for: A probiotic bacterium modulates antitumour γδ T-cell responses in lung cancer
Source: Front Immunol. 2026 Mar 24;17:1750569. doi: 10.3389/fimmu.2026.1750569 (PMC13079699; doi:10.3389/fimmu.2026.1750569)
Supplement: Supplementary file 1 [file DataSheet1.pdf]

Supplementary Figures 1-13

A probiotic bacterium modulates antitumour γδ T-cell responses in lung cancer

Yoshihiko Goto[1,2†], Garry Dolton[3†], Hannah Thomas[3], Théo Morin[3], Yuka Tajima[1,2], Kosuke Imamura[2], Shinya Sakata[2], Kentaro Oka[4], Atsushi Hayashi[4], Motomichi Takahashi[4], Takamasa Ueno[1], Takuro Sakagami[2], Yusuke Tomita[2*], Andrew K. Sewell[1,3,5‡*] and Chihiro Motozono[1‡]

1. Division of Infection and Immunity, Joint Research Center for Human Retrovirus infection, Kumamoto University, Kumamoto, Japan
2. Department of Respiratory Medicine, Faculty of Life Sciences, Kumamoto University, Kumamoto, Japan
3. Division of Infection and immunity Cardiff University School of Medicine, Cardiff, UK
4. R&D Division, Miyarisan Pharmaceutical Co., Ltd., Saitama, Japan
5. Systems Immunity Research Institute, Cardiff University, Cardiff, UK

[†]These authors contributed equally to this work and share first authorship
[‡]These authors contributed equally to this work and share last authorship

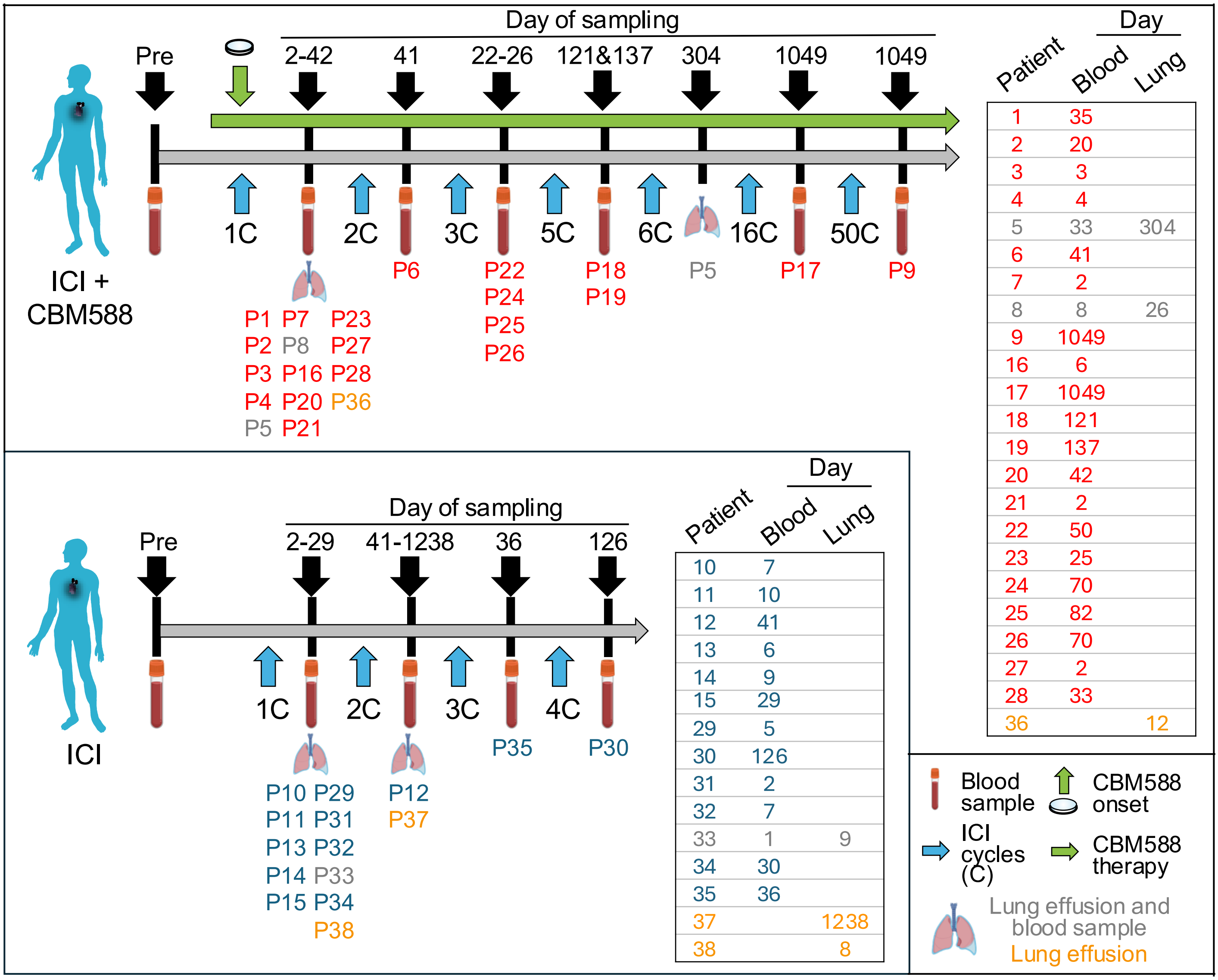

Patient	Day: Blood	Day: Lung
1	35	
2	20	
3	3	
4	4	
5	33	304
6	41	
7	2	
8	8	26
9	1049	
16	6	
17	1049	
18	121	
19	137	
20	42	
21	2	
22	50	
23	25	
24	70	
25	82	
26	70	
27	2	
28	33	
36		12

Patient	Day: Blood	Day: Lung
10	7	
11	10	
12	41	
13	6	
14	9	
15	29	
29	5	
30	126	
31	2	
32	7	
33	1	9
34	30	
35	36	
37		1238
38		8

Supplementary Figure 1. Patient overview. Patients treated with immune checkpoint inhibitors (ICI), with or without CBM588. Blood (pre and/or post ICI) and lung pleural effusions taken post ICI (days), as indicated by the time courses and tables. ICI regimen: PD-1, + CTLA-4 (P15), or PD-L1 (P1 and P5).

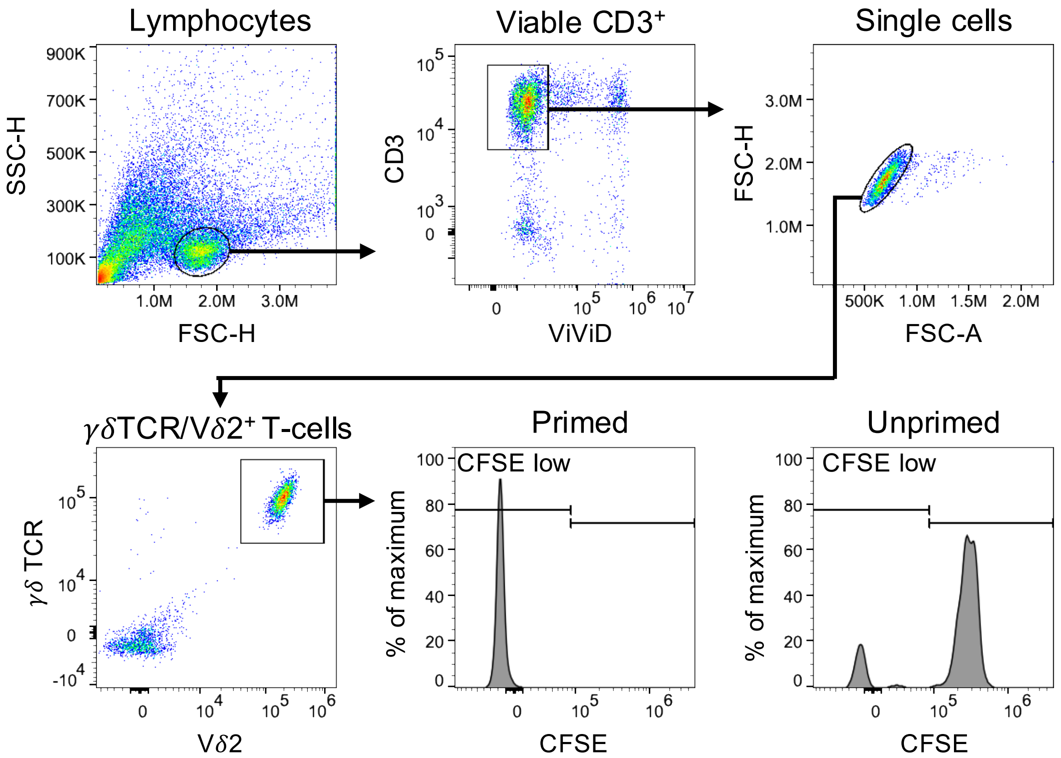

Supplementary Figure 2. Flow cytometry gating strategy for CFSE proliferation. Comparison of $CFSE^{low}$ (dividing cells) and $CFSE^{high}$ populations of primed and unprimed $\gamma\delta$ TCR^{+} $V\delta2^{+}$ T-cells.

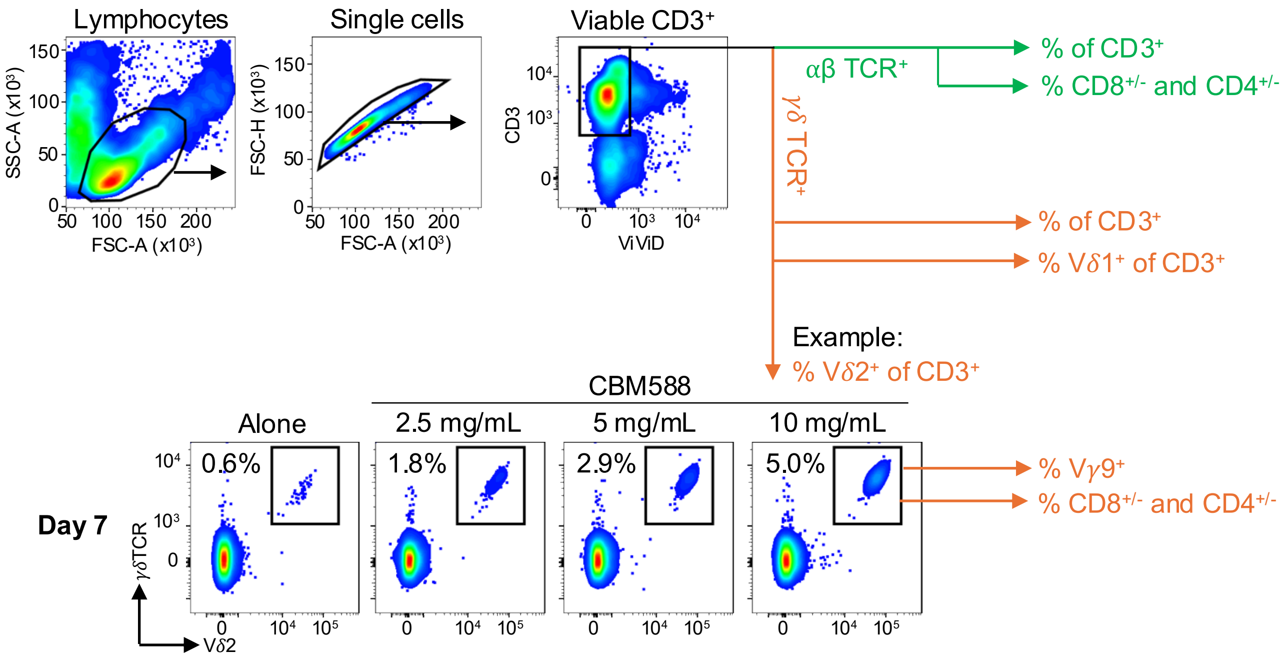

Supplementary Figure 3. Flow cytometry gating strategies A. Flow cytometry gating strategies for different T-cell subsets following the priming of PBMCs with CBM588 tablet at different concentrations. Flow cytometry data for $\gamma\delta$ TCR$^+$ Vδ2$^+$ T-cells shown as an example for one of the donors.

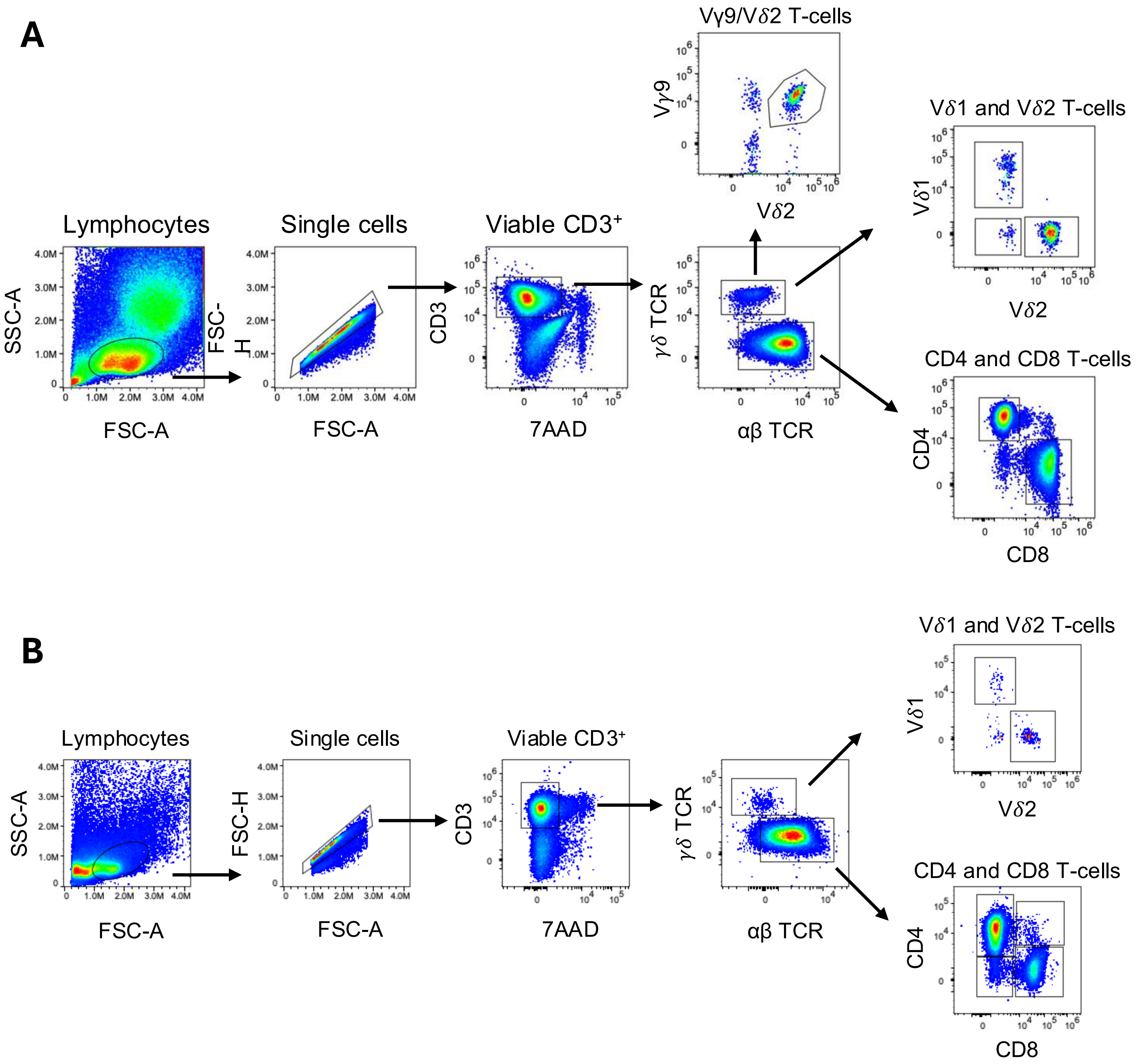

Supplementary Figure 4. Flow cytometry gating strategies. A. Flow cytometry gating strategy for various markers on αβ TCR$^+$ and $\gamma\delta$ TCR$^+$ T-cell subsets in PBMCs. **B.** Flow cytometry gating strategy for αβ TCR$^+$ and $\gamma\delta$ TCR$^+$ T-cell subsets in lung effusions from patients.

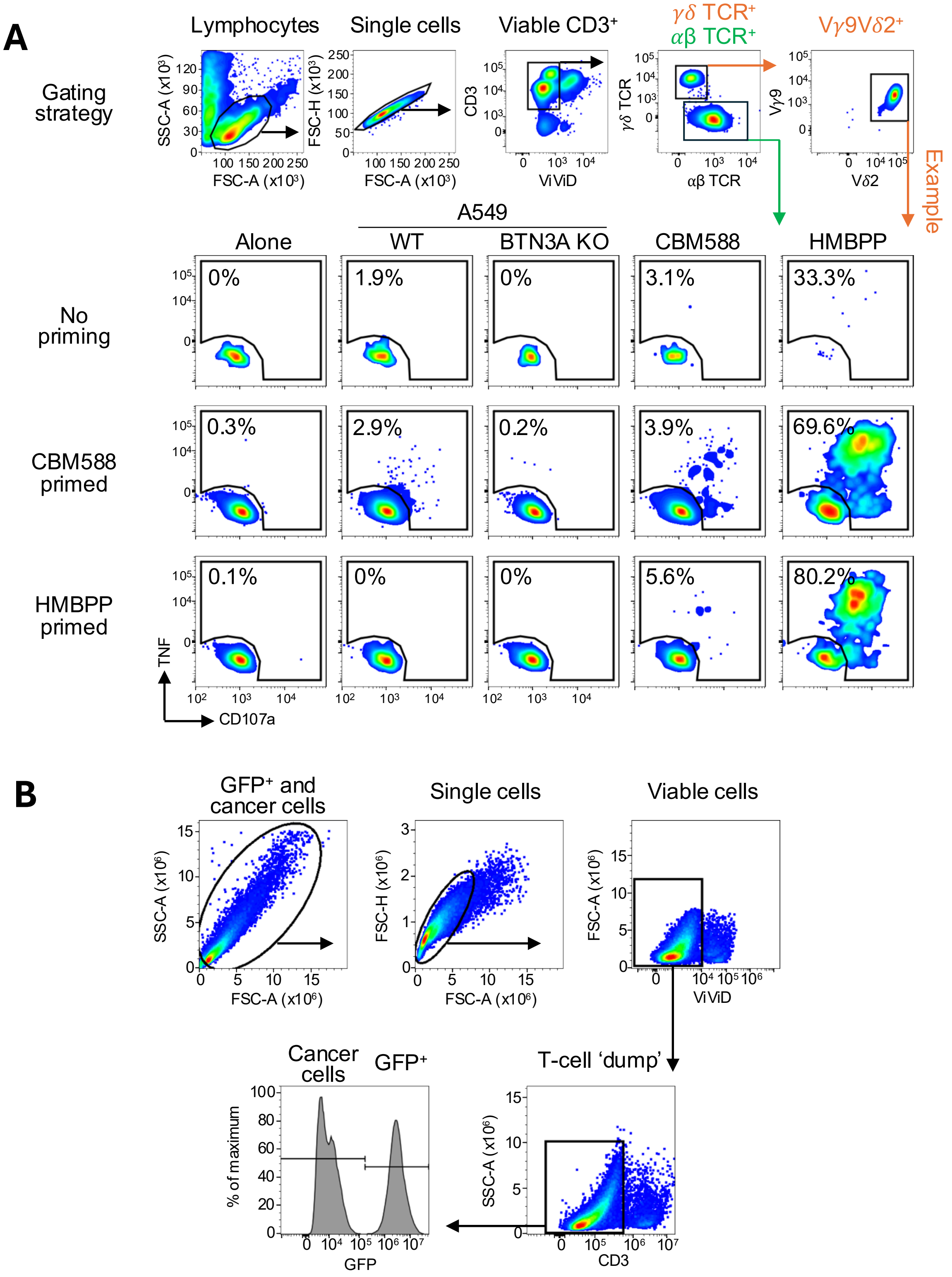

Supplementary Figure 5. Gating strategies for T107 assay and flow cytometry based killing assays. A. T107 assay flow cytometry gating strategy for functional testing of CBM588 tablet (10 mg/mL) or HMBPP (10 ng/mL) primed PBMCs from healthy donors. Example data set from a T107 assay for one healthy donor primed with CBM588 tablet or HMBPP, then tested against A549 cells, A549 BTN3A knockout cells, CBM588 tablet or HMBPP. Percentages shown for the TNF/CD107a^{+} gate. **B.** Flow cytometry gating strategy for killing assays. GFP^{+} reference cells added to assay wells immediately prior to harvest and staining for flow cytometry.

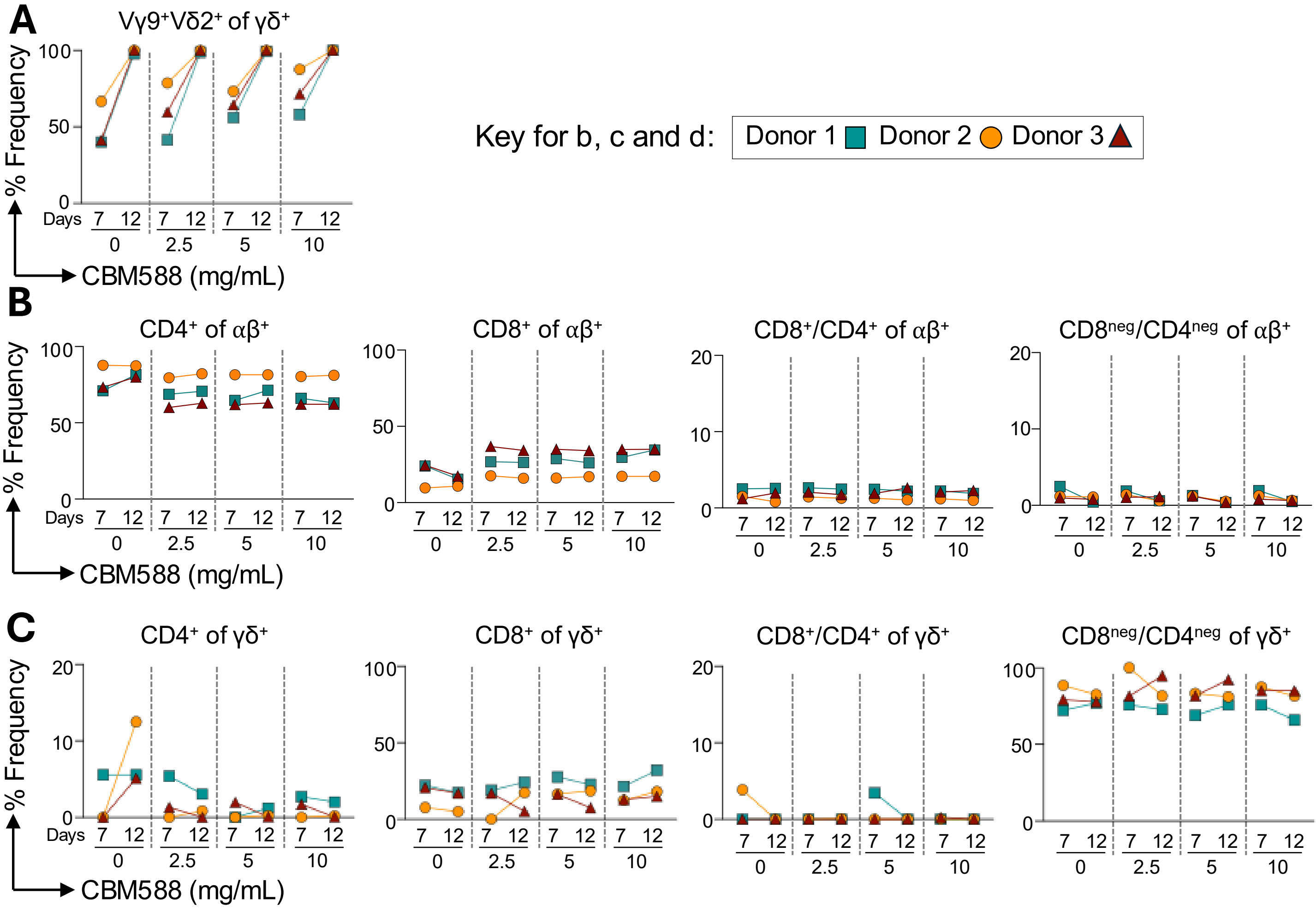

Supplementary Figure 6. T-cell dissection following priming with CBM588 tablet. A. Frequency of $\gamma\delta$ TCR$^+$ Vγ9$^+$ T-cells. **B**. Frequency of $\alpha\beta$ TCR$^+$ T-cells, with analysis based on co-receptor expression. **C**. Co-receptor dissection of $\gamma\delta$ TCR$^+$ T-cells.

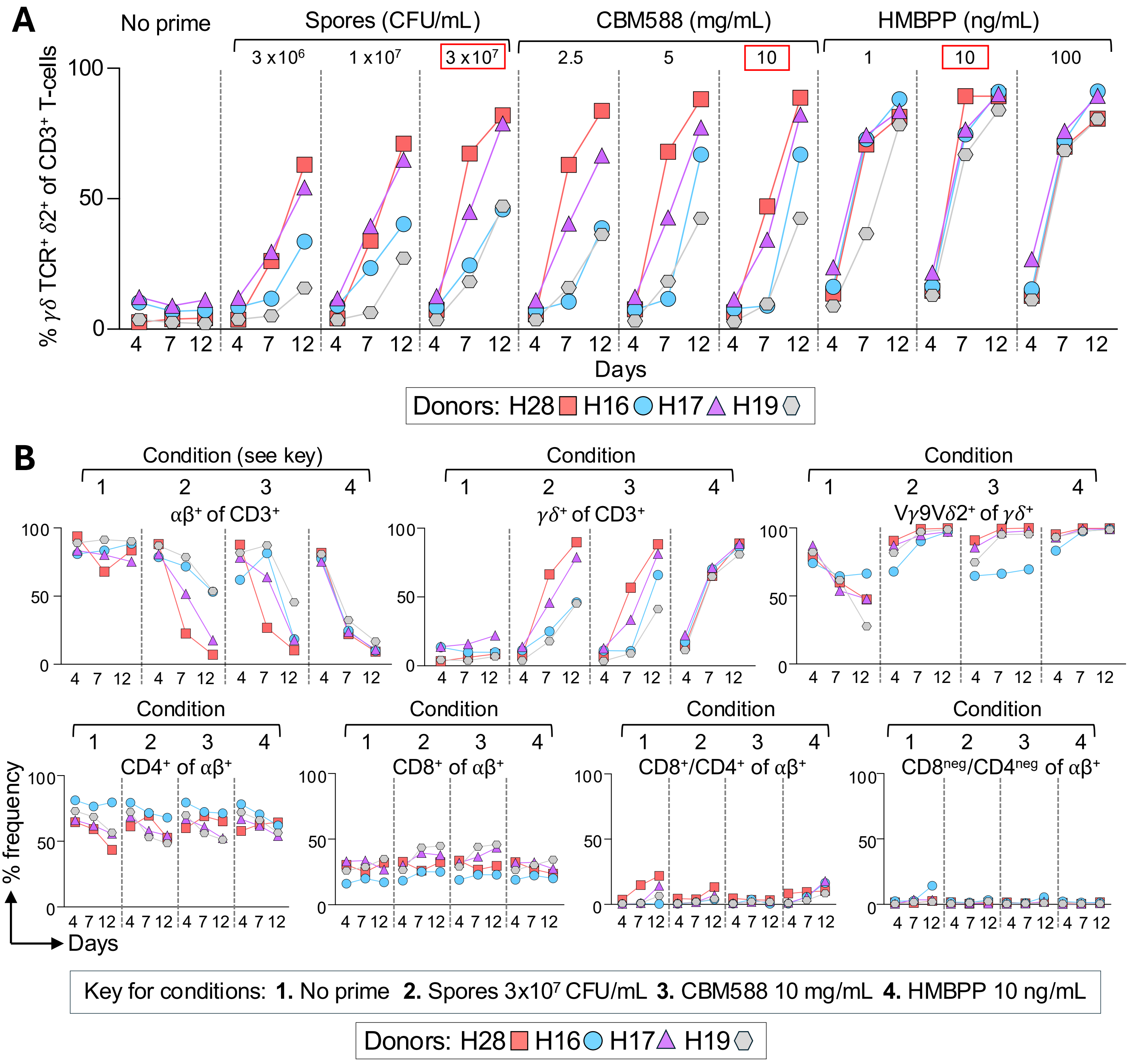

Supplementary Figure 7. Frequency of $\gamma\delta$ TCR$^+$ Vδ2$^+$ T-cells following stimulation with CBM588 spore, CBM588 tablet and HMBPP. A. Frequency of $\gamma\delta$ TCR$^+$ Vδ2$^+$ T-cells in CD3$^+$ T-cells after stimulation of PBMCs from four healthy donors with CBM588 spore, suspension of CBM588 tablet or HMBPP. T-cells sampled at days 4, 7 and 12 post stimulation. Data indicated by the red boxes, for colony forming units (CFU) of spore, and concentrations of CBM588 tablet and HMBPP, are also displayed in **Figure 1B**. **B**. Frequency of T-cell subsets after stimulation of PBMCs for the four healthy donors in (a) with CBM588 spores, suspensions of CBM588 tablet, and HMBPP at the CFUs or concentrations indicated in the key.

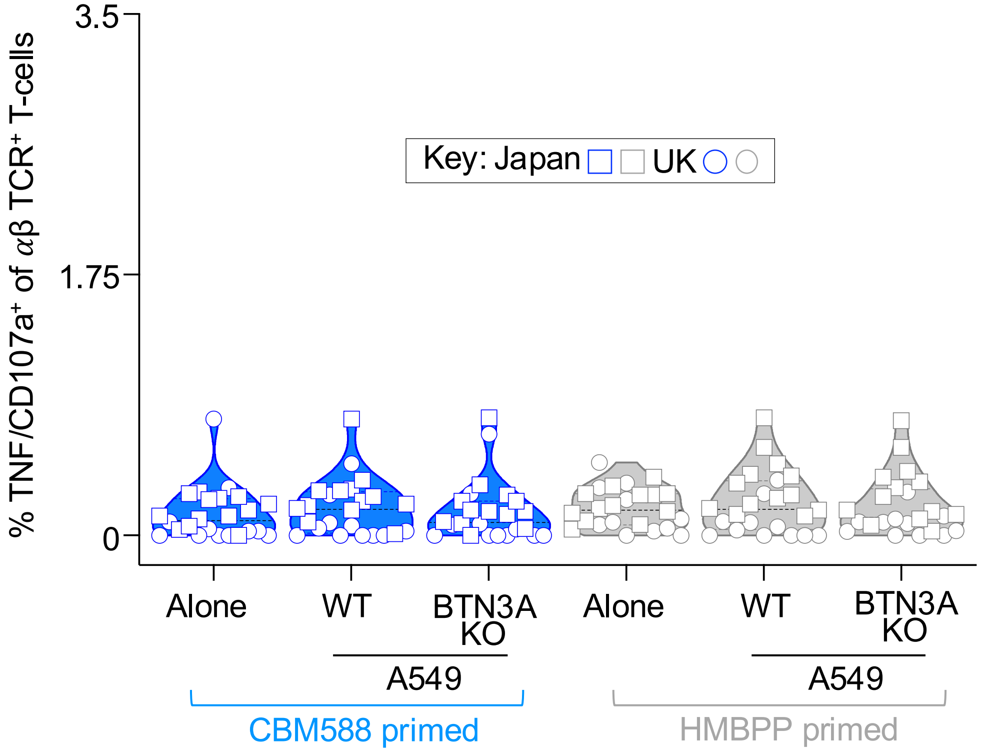

Supplementary Figure 8. Reactivity of CBM588 primed PBMCs against cancer cells. $\alpha\beta$ TCR^+ T-cell reactivity ($TNF/CD107a^+$) of CBM588 tablet (10 mg/mL) or HMBPP (10 ng/mL) primed PBMCs from healthy donors from the United Kingdom or Japan (indicated in key) towards A549 and A549 BTN3A knock out (KO) cells.

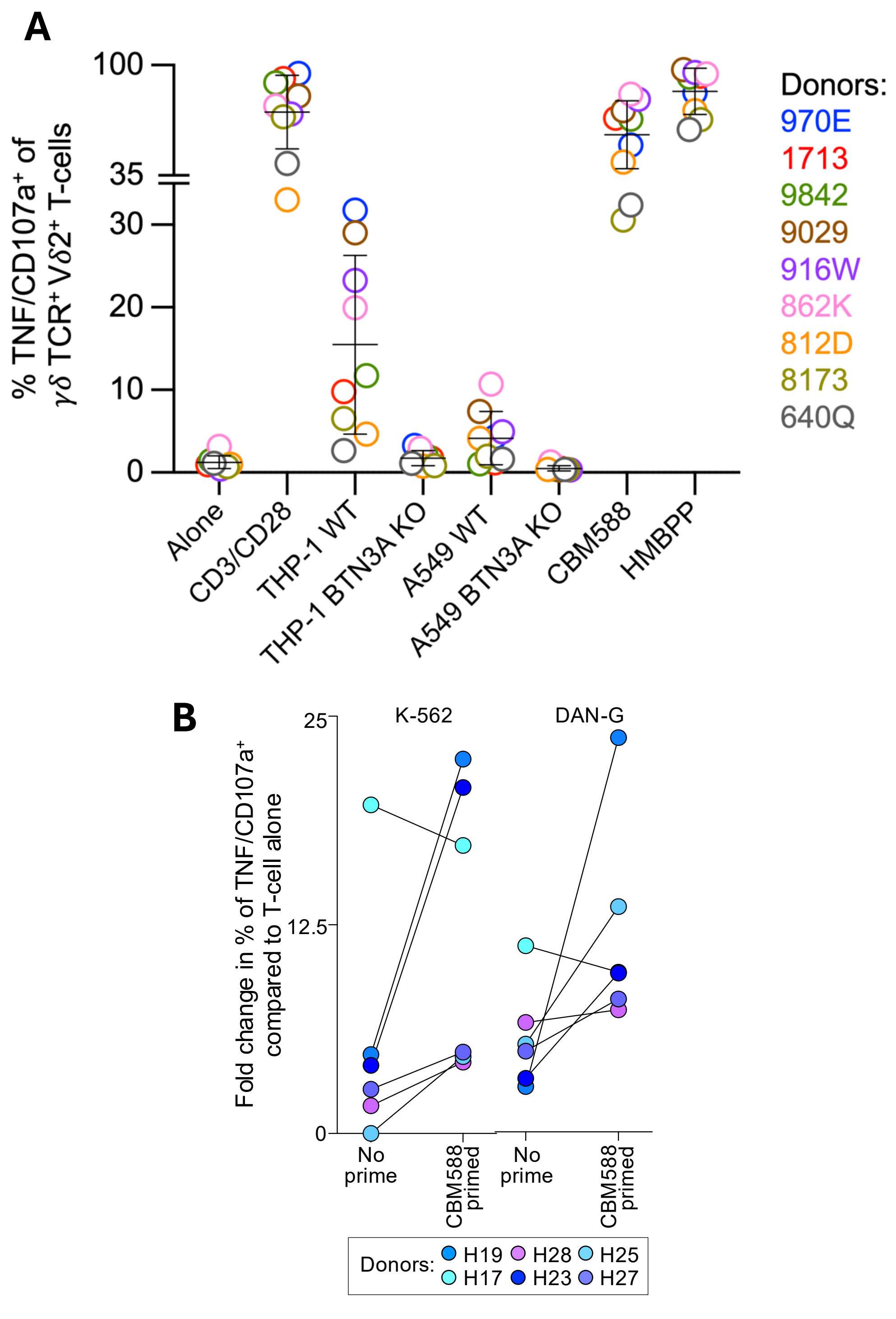

Supplementary Figure 9. CBM588 primed T-cell recognition of of various cancer types. A. CBM588 primed PBMCs from nine healthy donors tested in a T107 assay (4h) with CD3/CD28 DynaBeads, THP-1 cells +/- BTN3A, A549 cells +/- BTN3A, CBM588 tablet (10 mg/mL) and HMBPP (10 ng/mL). Gated on viable $CD3^{+}/\gamma\delta TCR^{+}/V\delta 2^{+}$ T-cells. **B**. T107 assay (TNF and CD107a) for six healthy donors primed with CBM588 tablet then tested against K-562 (leukemia) and DAN-G (pancreatic) cancer cells. Gated on $V\gamma 9V\delta 2^{+}$ T-cells. The fold increase in reactivity to cancer cells relative to T-cells alone is displayed.

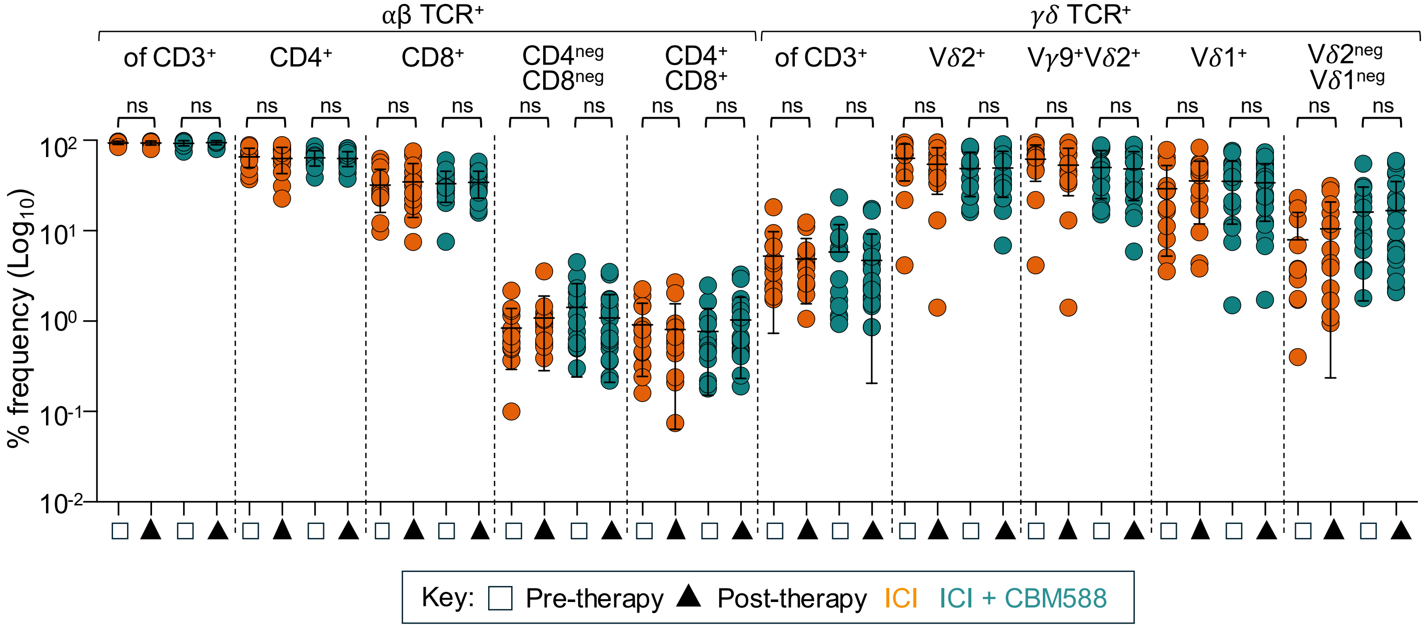

Supplementary Figure 10. Frequency of peripheral blood T-cell subsets from lung cancer patients receiving ICI or ICI and CBM588. Analysis of the frequency $\gamma\delta$ TCR$^+$ and αβ TCR$^+$ T-cell subsets with statistical comparisons made between pre- and post-therapy for immune checkpoint inhibitor (ICI) therapy alone or ICI combined with CBM588.

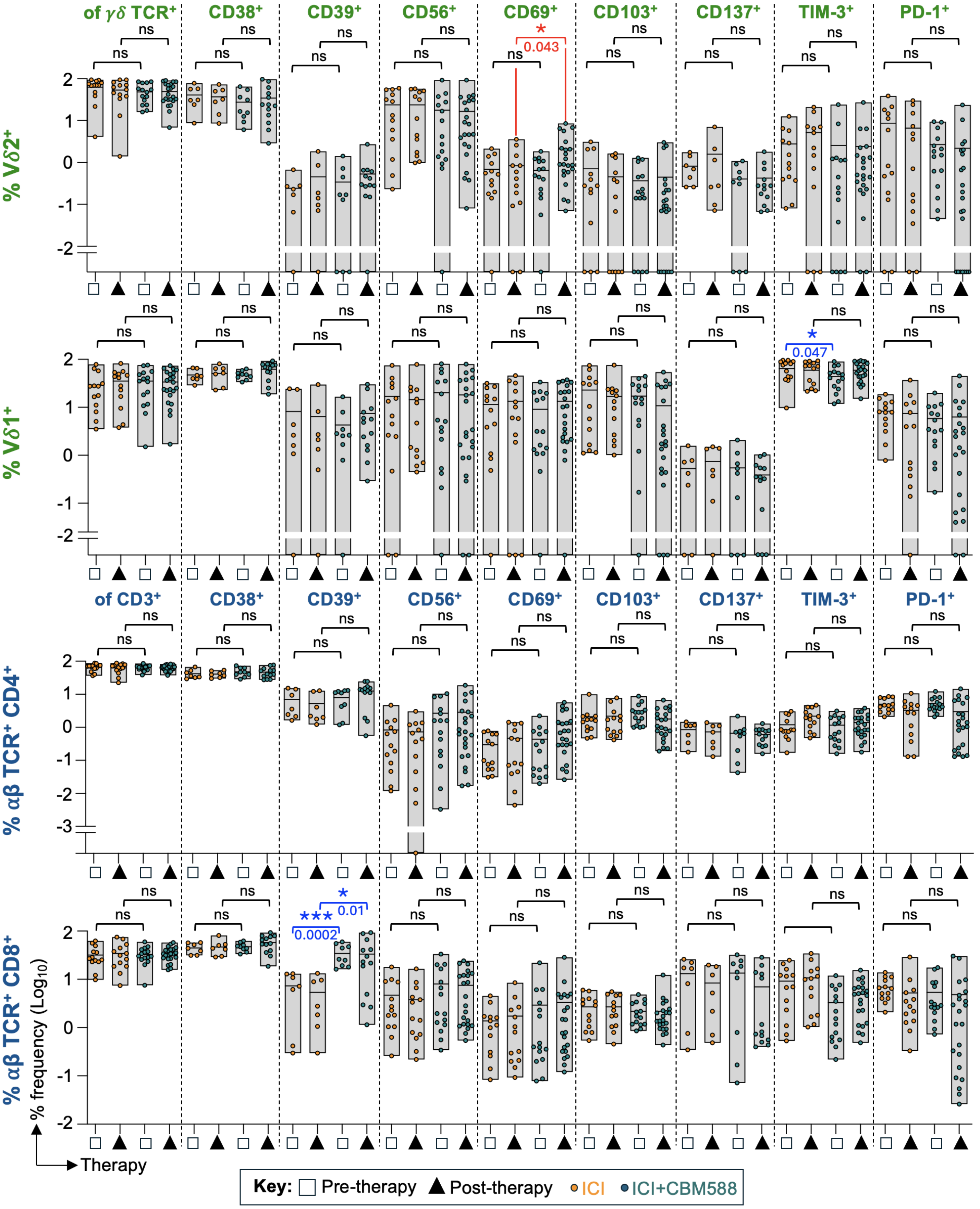

Supplementary Figure 11. Frequency and phenotype of peripheral blood T-cell subsets from lung cancer patients receiving ICI or ICI+CBM588. Analysis of the frequency and phenotype of various T-cell subsets, with statistical comparisons made between pre- and post-therapy for immune checkpoint inhibitor (ICI) therapy alone and ICI combined with CBM588. Notable findings include a significant difference in CD69 expression on Vδ2$^+$ T-cells post-therapy between ICI and ICI+CBM588 (red text), whereas no significant difference was observed pre-therapy (same data as in **Fig. 3B**). For CD39 expression on αβ TCR$^+$CD8$^+$ T-cells, a significant difference for post-therapy CD39 between ICI and ICI+CB588 was also seen before therapy (blue text). Additionally, TIM3 expression on Vδ1+ T-cells showed a significant difference only in the pre-therapy comparison between ICI and ICI+CBM588 (blue text). In summary, significant differences shown in blue text were present before therapy. The only significant difference present only post therapy is shown in red. P values are displayed for a two-tailed Mann-Whitney test.

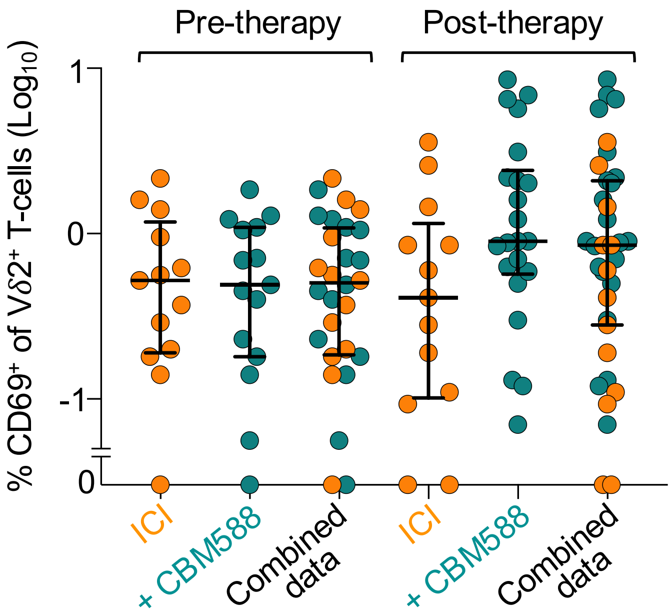

Supplementary Figure 12. CD69 expression of peripheral blood Vδ2^{+} T-cells from lung cancer patients treated with ICI or ICI+CBM588. CD69 expression on Vδ2^{+} T-cells from the blood of patients on ICI or ICI+CBM588, pre- and post-therapy. Median is displayed with error bars depicting the interquartile range. The same data is also displayed in **Fig. 3B** and shown here for combined display of the cohorts with the median.

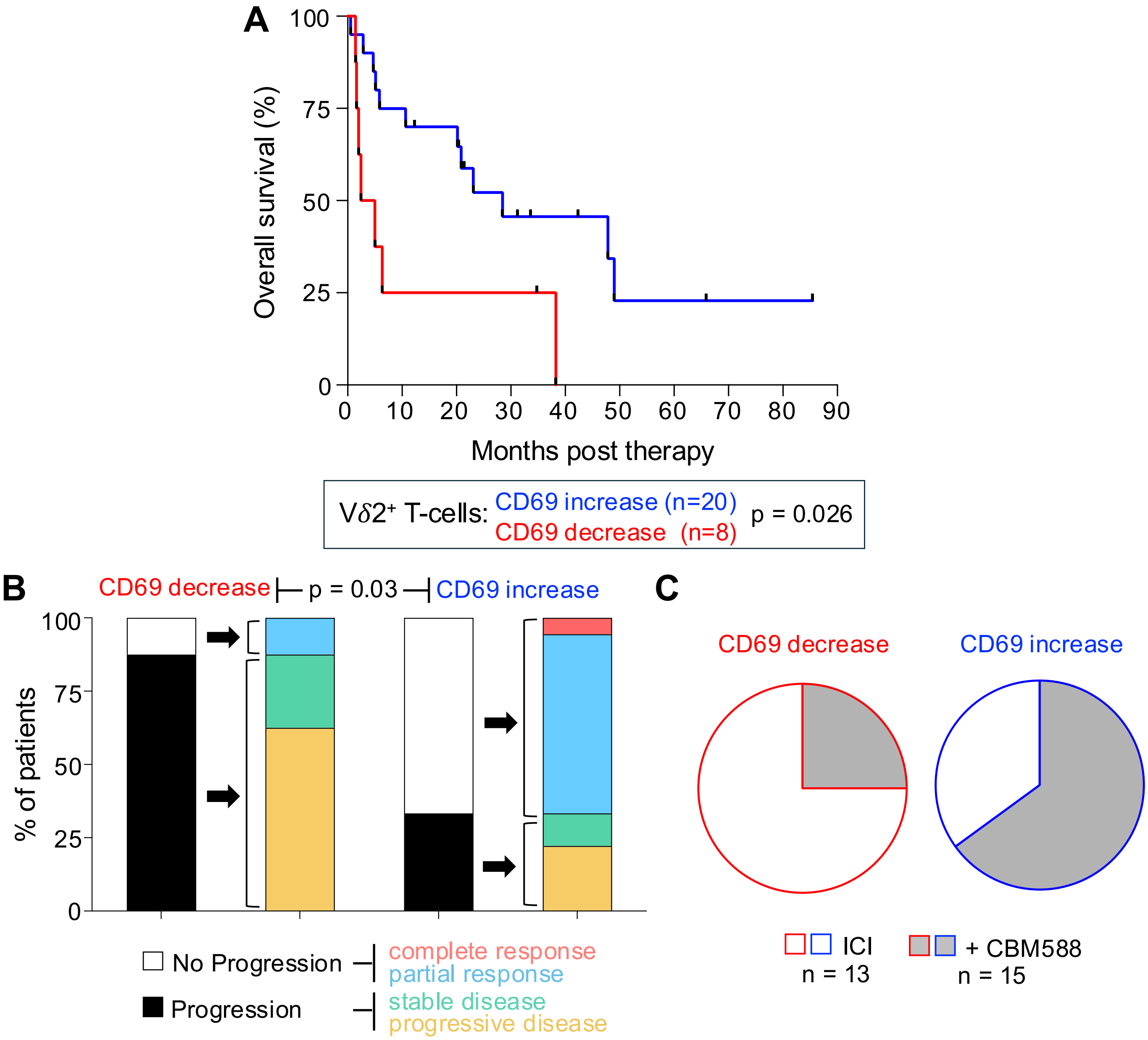

Supplementary Figure 13. An increase in CD69 expression on Vδ2$^+$ T-cells post-therapy correlates with improved outcomes in lung cancer patients receiving immunotherapy. A. Kaplan-Meier curve showing overall survival of lung cancer patients receiving either ICI or ICI+CBM588 (considered as one cohort, n = 28). Patients were categorized into two groups based on CD69 expression on Vδ2$^+$ T-cells: increase or decrease in CD69 post-therapy relative to pre-therapy. P value for a log-rank test. **B**. Decrease or increase in CD69 expression on Vδ2$^+$ T-cells categorized by clinical outcome according to the key. *p* value for Fisher's exact test. **C**. Patients on ICI or ICI + CBM588 within the CD69 decrease or increase categories.
